# Investigating the Molecular Basis of Retinal Degeneration in a Familial Cohort of Pakistani Decent by Exome Sequencing

**DOI:** 10.1371/journal.pone.0136561

**Published:** 2015-09-09

**Authors:** Bruno Maranhao, Pooja Biswas, Alexander D. H. Gottsch, Mili Navani, Muhammad Asif Naeem, John Suk, Justin Chu, Sheen N. Khan, Rachel Poleman, Javed Akram, Sheikh Riazuddin, Pauline Lee, S. Amer Riazuddin, J. Fielding Hejtmancik, Radha Ayyagari

**Affiliations:** 1 Department of Ophthalmology, University of California La Jolla, La Jolla, CA, United States of America; 2 The Wilmer Eye Institute, Johns Hopkins University School of Medicine, Baltimore, MD, United States of America; 3 National Centre of Excellence in Molecular Biology, University of the Punjab, Lahore, Pakistan; 4 Allama Iqbal Medical College, University of Health Sciences, Lahore, Pakistan; 5 National Centre for Genetic Diseases, Shaheed Zulfiqar Ali Bhutto Medical University, Islamabad, Pakistan; 6 OGVF branch, National Eye Institute, NIH, Bethesda, MD, United States of America; Hadassah-Hebrew University Medical Center, ISRAEL

## Abstract

**Purpose:**

To define the molecular basis of retinal degeneration in consanguineous Pakistani pedigrees with early onset retinal degeneration.

**Methods:**

A cohort of 277 individuals representing 26 pedigrees from the Punjab province of Pakistan was analyzed. Exomes were captured with commercial kits and sequenced on an Illumina HiSeq 2500. Candidate variants were identified using standard tools and analyzed using exomeSuite to detect all potentially pathogenic changes in genes implicated in retinal degeneration. Segregation analysis was performed by dideoxy sequencing and novel variants were additionally investigated for their presence in ethnicity-matched controls.

**Results:**

We identified a total of nine causal mutations, including six novel variants in *RPE65*, *LCA5*, *USH2A*, *CNGB1*, *FAM161A*, *CERKL* and *GUCY2D* as the underlying cause of inherited retinal degenerations in 13 of 26 pedigrees. In addition to the causal variants, a total of 200 variants each observed in five or more unrelated pedigrees investigated in this study that were absent from the dbSNP, HapMap, 1000 Genomes, NHLBI ESP6500, and ExAC databases were identified, suggesting that they are common in, and unique to the Pakistani population.

**Conclusions:**

We identified causal mutations associated with retinal degeneration in nearly half of the pedigrees investigated in this study through next generation whole exome sequencing. All novel variants detected in this study through exome sequencing have been cataloged providing a reference database of variants common in, and unique to the Pakistani population.

## Introduction

Inherited retinal degenerations (IRD) are among the best studied of genetic disorders. To date more than 250 genes and an additional 40 loci have been identified to be associated with retinal degenerations [1]. In some instances there is a one-to-one correlation of the gene and disease phenotype, while in other instances a broad genetic and phenotypic heterogeneity [2] has been observed. IRD phenotypes can be inherited in an autosomal dominant [3], autosomal recessive [4], X-linked [5], or mitochondrial traits [6]. Complicating this further, mutations in the same gene can cause either syndromic or non-syndromic retinal degeneration [7, 8].

Targeted sequencing and/or microarray-based studies identified the genetic basis of about 50% of the total IRD cases [9]. The availability of next generation sequencing (NGS) methodologies improved the ability to carry out comprehensive analysis and utilization of these technologies have improved identifying the genetic basis to nearly 70% of cases of IRD in certain populations [10–13]. Inbred populations provide an opportunity to investigate recessive genetic disorders. Pakistani population with high consanguinity offers an opportunity to investigate the genetic basis of inherited disease including IRD. A single study involving children at the Ida Rieu School for the blind and deaf concluded that roughly a third of blindness in Pakistan has a hereditary etiology [14]. Involvement of mutations in known retinal disease genes in causing IRD in Pakistani population has been previously described [4, 15–23]. In this study, we examined 26 pedigrees from the Punjab province of Pakistan using exome sequencing to determine the genetic basis of early onset retinal degeneration and the contribution of known gene mutations. These analyses also enabled identification of single nucleotide variants that are common in, and unique to the Pakistani population.

## Methods and Material

### Ethics Statement

The present study was approved by the Institutional Review Boards (IRB) of the National Centre of Excellence in Molecular Biology, Lahore Pakistan, the National Eye Institute, Bethesda MD, the Johns Hopkins University, Baltimore MD, and the University of California, San Diego. The study protocol is consistent with the tenets of the Declaration of Helsinki. Written informed consent was obtained from all participating subjects.

### Subjects

This study was approved by the institutional review boards (IRB) of National Centre of Excellence in Molecular Biology, Lahore Pakistan, and Johns Hopkins University, Baltimore MD. All participating family members have provided an informed written consent that has been endorsed by the respective IRBs and is consistent with the tenets of the Declaration of Helsinki.

A detailed clinical and medical history was obtained from the individual families. Selected affected as well as unaffected individuals underwent standard ophthalmic evaluation including fundus photography, Electroretinography, measurement of visual acuity and visual fields. Funduscopy was performed at Layton Rehmatulla Benevolent Trust (LRBT) Hospital (Lahore, Pakistan). Electroretinography (ERG) measurements were recorded by using equipment manufactured by LKC (Gaithersburg, MD). Dark-adapted rod responses were determined through incident flash attenuated by -25dB, whereas rod–cone responses were measured at 0 dB. The 30 Hz flicker responses were recorded at 0 dB to a background illumination of 17 to 34 cd/m^2^. Blood samples were collected from all available members and genomic DNA was extracted as previously described [24, 25].

**Fig 1:**
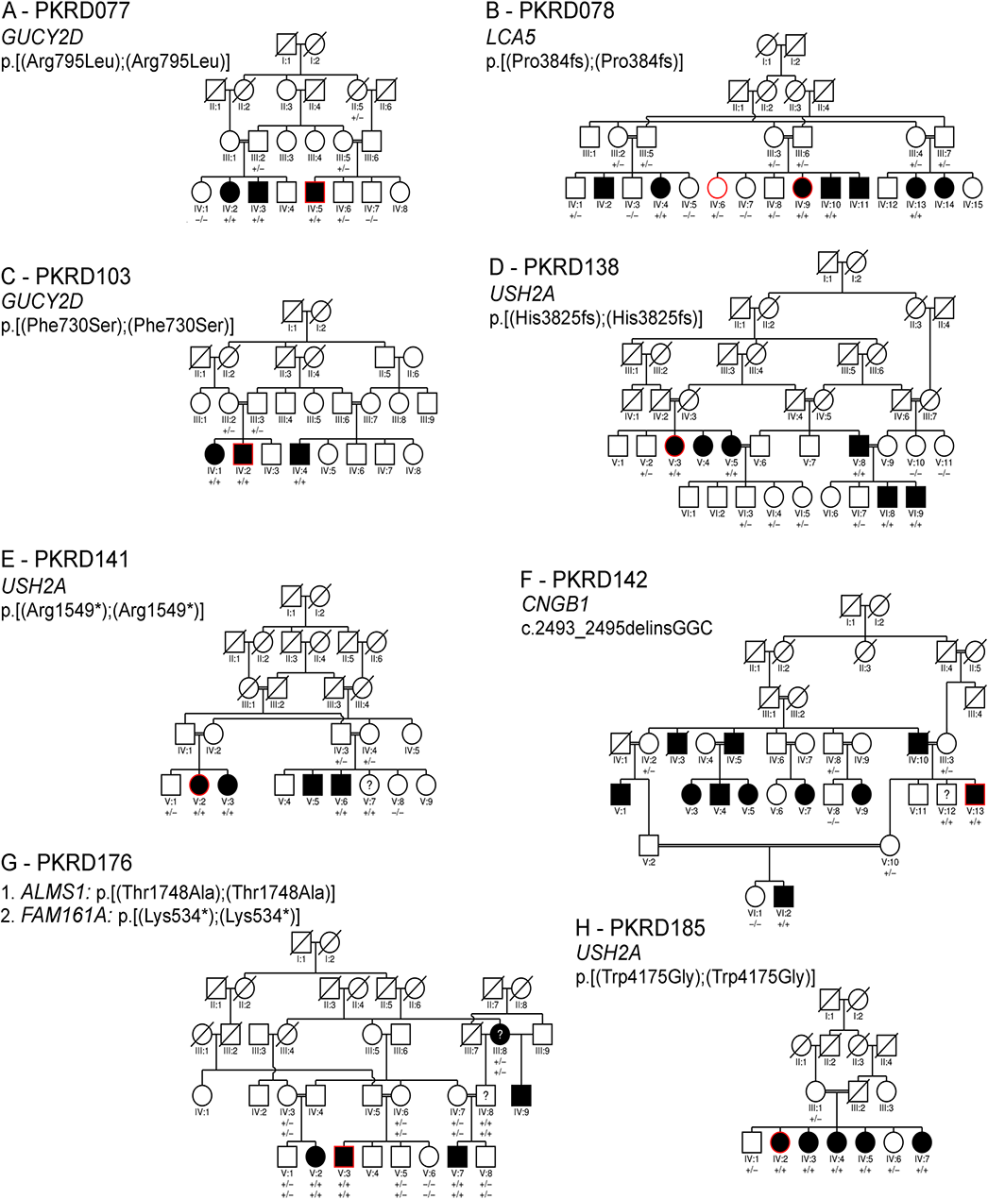
Members of the pedigree enrolled in this study are indicated by the presence of a genotype below their icon. “+” indicates presence of the variant, “-” indicates absence of the variant indicated to the left of the pedigree. Icons representing the individual(s) selected for exome capture and sequencing are outlined in red. A question mark (?) within the icon indicates the patient was not available for clinical evaluation. (A) PKRD077. (B) PKRD078. (C) PKRD103. (D) PKRD138. (E) PKRD141. (F) PKRD142. (G) PKRD176 (H) PKRD185.

**Fig 2:**
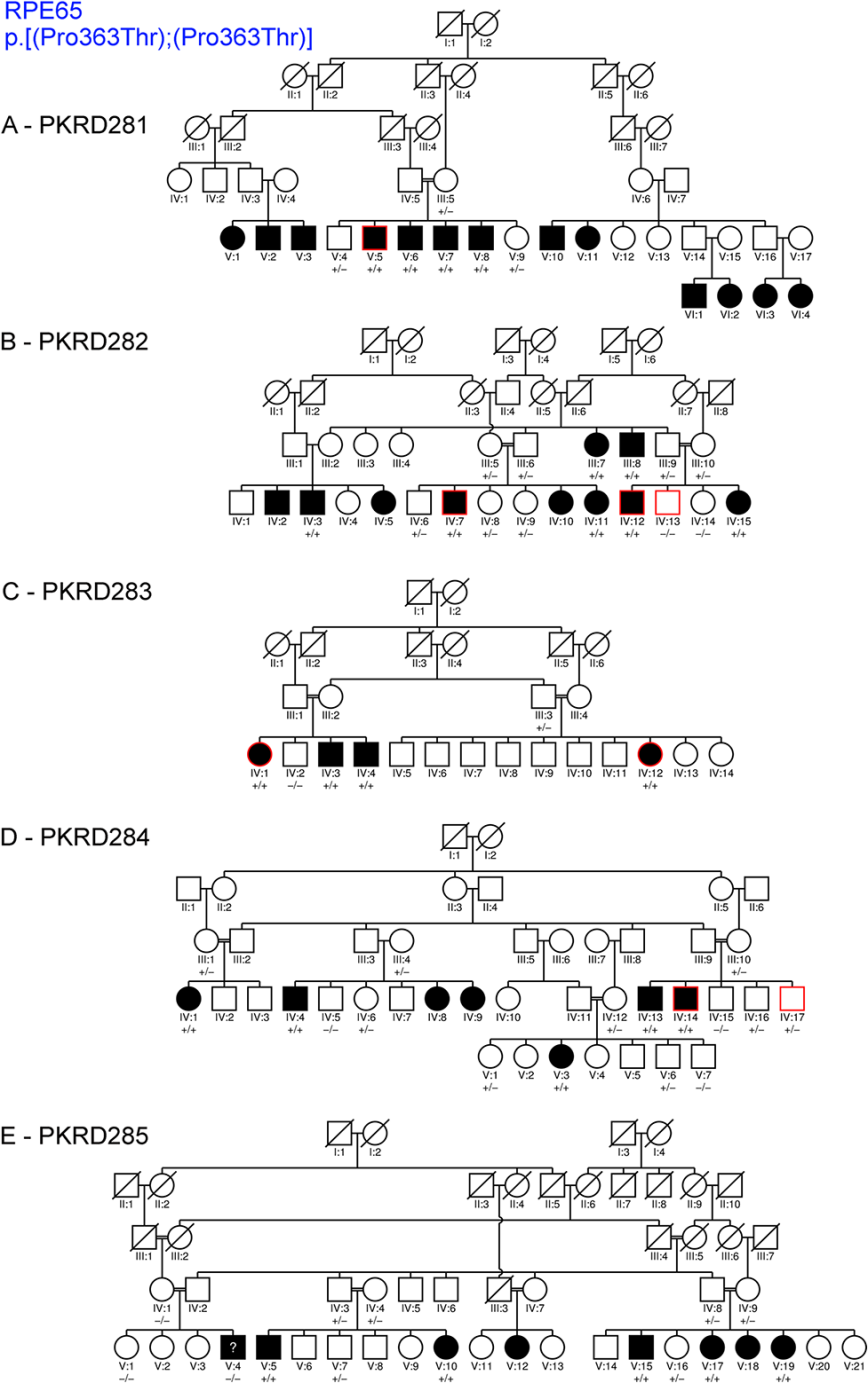
Members of the pedigree enrolled in this study are indicated by the presence of a genotype below their icon. “+” indicates presence of the variant, “-” indicates absence of RPE65 variant (NM_000329.2:c.1087 C>A, NP_000320.1:p.Pro363Thr). Icons representing the individual(s) selected for exome capture and sequencing are outlined in red. A question mark (?) within the icon indicates the patient was not clinically examined. (A) PKRD281. (B) PKRD282. (C) PKRD283. (D) PKRD284. (E) PKRD285.

A total of 26 consanguineous pedigrees were examined from the Punjab region of Pakistan. These pedigrees contain between 1–6 consanguineous marriages, and present 3–12 family members affected with early onset retinal degeneration. Of these, 5–16 members from each pedigree participated bringing the total to 277 individuals in 26 pedigrees (Figs 1 and 2).

### Exome Capture, Sequence Alignment, Variant Calling & Analysis

Sixty three individuals representing 25 of the 26 pedigrees were sequenced with one of the following exome capture kits: 9 samples with Nimblegen V2, 18 with Nimblegen V3, 7 with Agilent V5, 25 with Agilent V5+UTRs (S1 Table), and sequenced on an Illumina HiSeq 2500 as described earlier [26]. Reads were mapped with Novoalign to hg19; any reads that were not uniquely mapped were excluded from further analysis. PCR duplicates were removed with SAMtools, and variants were called with GATK.

Variants were annotated, filtered and prioritized for further analysis using exomeSuite [27]. The variants were first filtered for a recessive inheritance pattern since it was presumed the disease causative variant would be found in the homozygous state due to the consanguineous nature of the pedigrees examined. Variants were then filtered to eliminate: (1) all variant(s) with a homozygous genotype frequency of greater than 0.5% in HapMap [28], 1000 Genome [29], and/or NHLBI ESP6500 [30] databases, (2) all variants outside of a transcribed region, (3) all variants not residing within the six bases upstream of the start codon, (4) all variants not located within a miRNA targeted region as identified from the TargetScan [31] database, (5) all SNVs producing a missense change that was predicted to be benign by either MutationTaster [32], Polyphen [33], PROVEAN and SIFT [34, 35], (6) all intronic variants residing 15 or more bases from the nearest splice site, (7) all intronic variants residing within 15 bases of the nearest splice site but not predicted to alter splicing by NNSplice [36], and (8) all variants pertaining to a transcript whose biotype as defined by Ensemble is “nonsense mediated decay,” “retained intron,” “pseudogene” or whose transcript is 5’ and/or 3’ incomplete. We investigated variants within genes known to be associated with retinal degeneration and passed the above filtering criteria [1] (S2 Table).

Targeted mutation screening for RPE65 c.1087 C>A (NM_000329.2) variant was performed on Pedigree PKRD285 by Sanger dideoxy sequencing. Segregation of variant(s) with the disease phenotype in all pedigrees was verified by Sanger dideoxy sequencing using primers listed in S3 Table. Novel variants segregating with the disease phenotype in their respective pedigree were also screened for their presence in 96 ethnically matched unrelated controls.

All single nucleotide variants with a read depth >10, mapping quality score >30, and not exhibiting strand bias during sequencing were tabulated by pedigree (S4 Table). All variants present in >5 pedigrees and absent from the dbSNP, the 1000 genomes and the UCSC database were cataloged.

## Results

**Fig 3:**
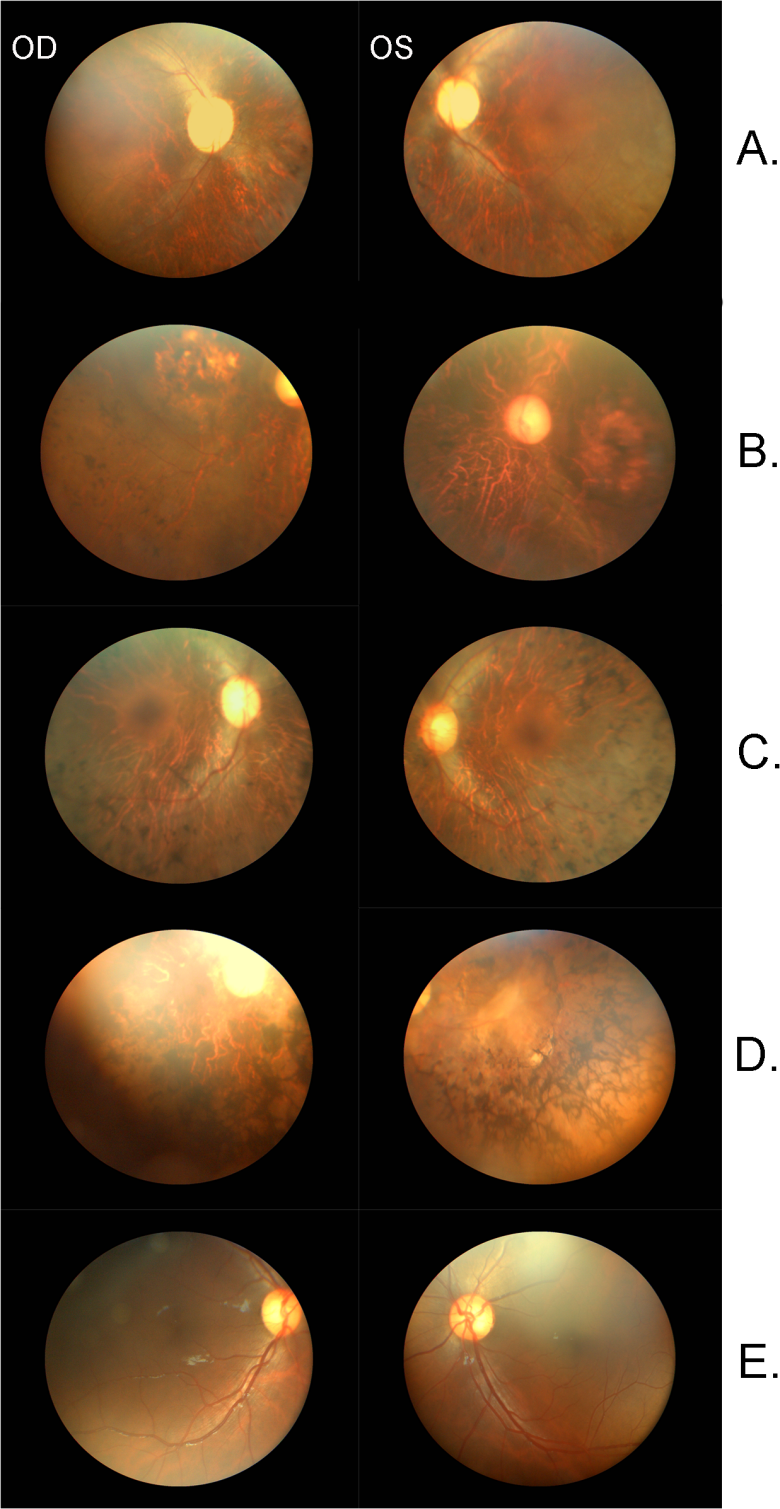
Fundus photographs of affected individuals with inherited retinal degeneration. Fundus photographs of the right and left eyes, respectively of affected individuals of families A) PKRD138, B) PKRD141, C) PKRD142 D) PKRD176 and E) an unaffected normal individual. Fundus photographs of the affected individuals show bone spicule-like pigmentation in the mid-periphery along with severe retinal attenuation.

**Fig 4:**
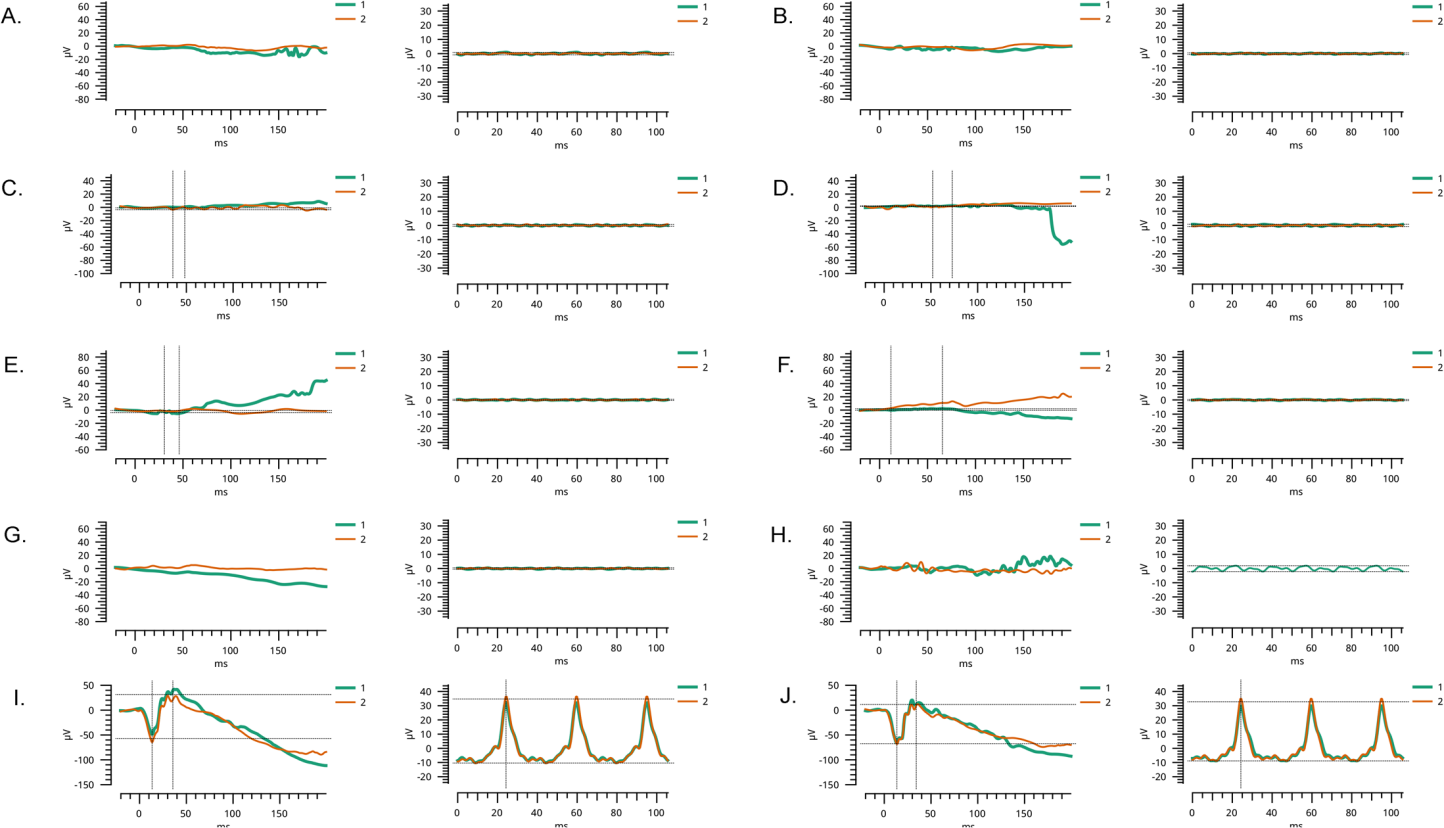
Electroretinography recordings of affected individuals with inherited retinal degeneration. Scotopic 0 dB response, and photopic 0 dB 30Hz flicker response of A) OD and B) OS of individual VI:9 of family PKRD138; C) OD and D) OS of individual V:5 of family PKRD141; E) OD and F) OS of individual VI:2 of family PKRD142; G) OD and H) 374 OS of individual V:7 of family PKRD176; I) OD and J) OS of an unaffected control. The electroretinography responses of affected individuals illustrate loss of rod and cone response. OD = oculus dexter (right eye); OS = oculus sinister (left eye).

In an ongoing effort to understand the molecular basis of inherited retinal degeneration, we investigated 25 large highly inbred familial cases to identify the causal variants through next-generation whole exome sequencing. Affected individuals in these familial cases experienced night blindness and decreased visual acuity in their early childhood. Medical records and clinical reports of earlier examinations were suggestive of retinal dystrophy with early, most likely congenital onset. Fundus photographs of the affected individuals show bone spicule-like pigmentation in the mid-periphery and arteriolar attenuation (Fig 3) along with severely diminished rod and cone response in electroretinography examination (Fig 4).

Whole exome sequencing of members of 25 Pakistani pedigrees generated on average 8.7 gigabases of data for each exome with an average coverage depth of 63 within the targeted region (S4 Table). Filtering sequence variants using specified criteria identified one to seven candidate variants in 13 pedigrees (S5 Table). A total of 2,904 single nucleotide variants were identified in five or more presumably unrelated pedigrees, and absent from the HapMap, the 1000 Genome and NHLBI ESP6500 databases. Of these, 200 were unreported in dbSNP and believed to be unique to the Pakistani population (S6 Table).

**PKRD077:** Sequencing the exome of individual IV: 5 and subsequent analysis of variants identified a homozygous non-synonymous coding variant c.2384 G>A (NM_000180.3) in *GUCY2D* that fulfilled the filtering criteria used to identify genes implicated in causing IRD (Fig 1A) and segregated with the IRD in PKRD077. The respective amino acid substitution: p.R795Q (NP_000171.1) in GUCY2D is predicted to be damaging by all four variant predictors algorithms used and has been reported previously [37]. An additional synonymous variant in *COL11A1* predicted not to affect splicing was also observed in IV: 5 (Table 1 and S5 Table).

**Table 1:** List of potentially causative variants segregating with retinal degeneration phenotype in Pakistani pedigrees.

Pedigree	Gene	cDNA Change	Protein Change	Novel/Known
PKRP176	ALMS1	NM 015120.4:c.5242 A>G	NP 055935.4:p.T1748A	Novel
PKRP142	CNGB1	NM_001297.4:c.2493-2_2495delinsGGC	Unknown	Novel
PKRP176	FAM161A	NM_001201543.1:c.1600A>T	NP_001188472.1:p.Lys534Ter	Novel
PKRP103	GUCY2D	NM_00180.3:c.2189T>C	NP_00171.1:p.Phe730Ser	Novel
PKRP077	GUCY2D	NM_00180.3:c.2384G>T	NP_00171.1:p.Arg795Leu	[37]
PKRP078	LCA5	NM_181714.3:c.1151delC	NP_859065.2:p.(Pro384fs)	[16]
PKRP281, 282, 283, 284, 285	RPE65	NM_000329.2:c.1087C>A	NP_000320.1:p.Pro363Thr	[38]
PKRP138	USH2A	NM_206933.2:c.11473del	NP_996816.2:p.(His3825fs)	Novel
PKRP141	USH2A	NM_206933.2:c.4645C>T	NP_996816.2:p.Arg1549Ter	Novel
PKRP185	USH2A	NM_206933.2:c.12523T>G	NP_996816.2:p.Trp4175Gly	Novel

**PKRD078:** In this sixteen-member pedigree with three consanguineous marriages, exomes of an affected (IV: 9) and an unaffected sibling (IV: 2) were sequenced (Fig 1B). A previously reported deletion mutation in *LCA5*: c.1151delC (NM_001122769.2, p.P384fs (NP_001116241.1) was identified that segregated with IRD phenotype in PKRD078 (Table 1 and S5 Table).

**PKRD103**: The exome of a single affected individual (IV:2) was sequenced in this four generation consanguineous pedigree (Fig 1C). Exome variant analysis identified a single novel homozygous non-synonymous coding variant in GUYC2D: c.2189 T>C (NM_000180.3:c.2189 T>C, NP_000171.1: p.F730S) that is predicted to be damaging. This novel variant segregated with disease in PKRD103 and was not detected in ethnicity matched controls (Table 1 and S5 Table).

**PKRD138**: Affected individual V:3 of this six generation pedigree was selected for exome sequencing (Figs 1D, 3 and 4). Analysis of exome variants identified a novel homozygous single base deletion in USH2A c.11473delC (NM_206933.2) predicted to result in a frame shift p.His3825fs was found to segregate with IRD. Analysis of Pakistani control DNA identified this to be a rare sequence alteration in the Pakistani population (Table 1 and S5 Table).

**PKRD141:** A single affected individual (V:2) from this five generation pedigree with four consanguineous marriages was selected for exome capture and sequencing (Figs 1E, 3 and 4). Two candidate variant that fulfilled the filtration criteria were identified: a nonsense variation in USH2A (NM 206933.2): c.4645 C>T, predicted to result in a premature termination: p.R1549*, and a missense variant in *BEST1* (NM_001139443.1:c.1633 C>A, NP_001132915.1:p.P545T) predicted to be damaging by PROVEAN and MutationTaster, but benign by SIFT and Polyphen. Dideoxy sequencing of all available members of PKRP141 confirmed segregation of the *USH2A* variant, but not the *BEST1* variant (Table 1 and S5 Table).

**PKRP142:** In a six generation pedigree with eight consanguineous marriages, one individual (V:13) was selected for exome capture and sequencing (Figs 1F, 3 and 4). Analysis of the exome variants detected two homozygous changes in known IRD genes. One of them was a non-synonymous coding variant in *CEP164* c.1702 A>C (NM_014956.4: p.T568P) predicted to be benign by PROVEAN, SIFT and MutationTaster, but damaging by Polyphen. And the second variant is an insertion-deletion in *CNGB1* (NM_001297.4:c.2493- 2_2495delinsGGC, p.S831fs). Dideoxy sequencing of nine members of PKRP142 confirmed segregation of the *CNGB1* variant, but not the *CEP164* variant with the disease phenotype (Table 1 and S5 Table).

**PKRP176:** This is a five-generation Pakistani pedigree involving three consanguineous marriages (Figs 1G, 3 and 4). Sequencing the exome of one individual (V:3) identified a novel homozygous nonsense variant in *FAM161A* (NM_001201543.1:c.1600 A>T,:p.K534*) and two non-synonymous homozygous coding missense variants: *ALMS1* (NM 015120.4:c.5242 A>G,.4:p.T1748A) predicted to be damaging by Polyphen, PROVEAN and SIFT, but not by MutationTaster, and RAX2 (NM_032753.3:c.236 G>A,:p.R79Q) predicted to be damaging by all algorithms used in this study. Dideoxy sequencing of 12 members of this pedigree confirmed segregation of the *FAM161A* and *ALMS1* variants, but not the *RAX2* variant with IRD (Table 1 and S5 Table).

**PKRP185:** The exome of a single affected individual (IV:2) was selected for exome capture and sequencing (Fig 1H). Two non-synonymous variants satisfied the filtering criteria. The first one, a c.12523 T>G change in *USH2A* (NM 206933.2: p.W4175G) was predicted to be damaging by all algorithms. And the second, in *ABHD12* (NM_001042472.2:c.1045 G>A, 1:p.A349T) was predicted to be damaging by MutationTaster and Polyphen, but not by PROVEAN and SIFT. Dideoxy sequencing confirmed segregation of the *USH2A* variant, but not the *ABHD12* variant with disease phenotype (Table 1 and S5 Table).

**PKRP281:** A single affected member (V:5) of this pedigree was selected for exome capture and sequencing (Fig 2A). Two non-synonymous coding variants c.1087 C>A (NM 000329.2: p.P363T) in RPE65, and second, a c.2612 C>G (NM_000214.2): p.P871R) in *JAG1* were identified based on the filtering criteria used. Dideoxy sequencing of the seven PKRP281 members confirmed segregation of the *RPE65* variant but not the *JAG1* variant with the disease phenotype (Table 1 and S5 Table).

**PKRD282**: An affected and unaffected sibling pair (IV: 12 and IV: 13, respectively) and affected individual (IV: 7) were selected for exome capture and sequencing from this four generation pedigree (Fig 2B). Similar to PKRP281, exome analysis identified a variant in *RPE65* c.1087 C>A (NM_000329.2, p.P363T) as the causal mutation segregating with the IRD in the family PKRD282 (Table 1 and S5 Table).

**PKRP283:** Two affected individuals (IV:1 and IV:12) were selected for exome capture and sequencing from this four generation pedigree involving two consanguineous marriages (Fig 2C). Similar to PKRD281 and PKRD282, exome analysis identified a variant in *RPE65* c.1087 C>A (NM_000329.2, p.P363T) as the causal mutation segregating with the IRD in the family PKRD282 (Table 1 and S5 Table).

**PKRP284:** An affected individual and his unaffected sibling (IV: 14 and IV: 17, respectively) from this five generation pedigree with three consanguineous marriages were selected for exome capture and sequencing (Fig 2D). Analysis of variants detected two non-synonymous coding variants that satisfied the filtering criteria. One of them is a variant in *CDH23* c.127 G>A (NM_001171933.1; p.V43I). The second one is the *RPE65* c.1087 C>A (NM_000329.2, p.P363T) variant that is detected in PKRD281, PKRD282 and PKRD283 pedigrees. Sanger dideoxy sequencing confirmed segregation of *RPE65* variant, but not the *CDH23* variant with the disease phenotype (Table 1 and S5 Table). The RPE65 variant has been previously reported as a causative mutation[38].

**PKRD285:** Subsequent to identification of the *RPE65* c.1087 C>A (NM_000329.2, p.P363T) variant in PKRD281, PKRD282, PKRD283 and PKRD284 pedigrees, targeted mutation screening for this variant was performed on PKRD285. This analysis revealed that the *RPE65* c.1087 C>A variant segregated with the IRD phenotype in this five generation pedigree with six consanguineous marriages (Fig 2E).

Analysis of exome sequence variants in remaining 13 large consanguineous pedigrees using the specified filtering criteria did not reveal potential pathogenic changes in known IRD associated genes.

In summary, analysis of exome variants of 25 consanguineous Pakistani pedigrees identified nine unique homozygous causal variants in seven genes that are known to be associated with a retinal degeneration phenotype. All candidate variants identified in each pedigree are listed in S5 Table.

## Discussion

Here, we report next-generation sequencing based genetic analyses of 26 consanguineous pedigrees of Pakistani decent that identified a total of nine causal mutations, which include six novel variants, in *RPE65*, *LCA5*, *USH2A*, *CNGB1*, *FAM161A*, *CERKL* and *GUCY2D* as the underlying cause of IRD in 13 of 26 pedigrees. In addition to the nine causal variants, 200 variants that were absent from the dbSNP, the 1000 genomes and the UCSC databases were identified. Each of these 200 variants was detected in five or more unrelated pedigrees investigated in this study suggesting that they are common in, and unique to the Pakistani population. A catalog of these novel variants provides a reference to variants common in, and unique to the Pakistani population.

Consistent with the phenotype observed in the cohort of patients analyzed in this study, the *RPE65*, *LCA5*, *USH2A*, *CNGB1*, *FAM161A*, *CERKL* and *GUCY2D* genes consisting mutations in 13 of the 26 pedigrees have been implicated in causing recessive non-syndromic retinal degeneration [1]. All novel causative variants detected in this study result in truncation of the protein or located in highly conserved domains. Mutations reported so far in *FAM161A* are either nonsense or frame-shift changes [39–41]. In pedigree PKRD176, a novel nonsense mutation, NM_001201543.1:c.1600A>T (NP_001188472.1:p.K534*), causing premature termination of the longer of two protein coding transcripts for *FAM161A* was identified. This transcript was observed to be the minor transcript in a human retinal cDNA library [41]. However, the Ocular Genomics Institutes Retinal Transcriptome data [42] suggested the opposite (169 reads that contain portions of the 168 base exon versus 63 that bridge it). Therefore the impact of the p.K534* variant in retinal tissue is unclear. In addition, a second novel variant in *ALMS1* (NM_015120.4:c.5242A>G; NP_055935.4:p.T1748A) predicted to be damaging by Polyphen, PROVEAN and SIFT, but not by MutationTaster was also found to segregate with disease in this pedigree (Fig 1G). Mutations in *FAM161A* are reported to be associated with early onset recessive retinitis pigmentosa while mutations in *ALMS1* are associated with Alstrom syndrome and non-syndromic Leber’s congenital amaurosis [7, 8]. The *FAM161A* and *ALMS1* genes are located 10 Mb apart on chromosome 2 and the novel variants observed in these genes may exist in linkage disequilibirium in pedigree PKRD176. The *ALMS1* variant is listed in the ExAC database with an allele frequency of 0.0023, where as the frequency of *FAM161A* variant is 0.0000088. While the nonsense mutation in *FAM161A* may be sufficient to cause retinal pathology in this pedigree, additional studies are needed to establish if the early onset retinal degeneration phenotype observed in this pedigree is due to one or both of the potentially pathogenic variants segregating in this pedigree.

Due to the high consanguinity in Pakistani population, involvement of a smaller number of common disease causing mutations might be expected in pedigrees with IRD from a single province in Pakistan. Consistent with this, a single *RPE65* mutation was observed in five out of 26 pedigrees studied. However, the remaining 8 mutations are detected in single pedigrees. Among the nine total mutations detected in 13 pedigrees, seven causative variants were not reported previously in Pakistani patients. Furthermore, identification of six novel causative variants in known IRD genes emphasizes the understudied nature of this population. These novel mutations will enhance our Additionally, discovery of novel potentially disease causing variants in well-investigated retinal disease genes will expand our understanding of their role in ocular tissue and the disease mechanism. Although a limited number of pedigrees were investigated in this study, the identification of causal mutations in 13 out of 26 pedigrees accounting for 50% of the cases is consistent with similar observations on the contribution of known IRD gene mutations in other populations [10–13, 43].

An inherent advantage of next-generation based exome analysis is the identification of all potential variants in the exome in addition to the causative mutation that might act as modifiers of the disease phenotype. Consistent with this, presence of additional potentially pathogenic sequence alterations in IRD genes was observed in the set of pedigrees analyzed (S5 Table). Detailed and systematic analysis of the phenotype and genotype of patients in these pedigrees may elucidate the potential role of additional pathogenic variants as genetic modifiers.

In all but four pedigrees investigated in this study, sequencing was carried out on probands alone. The results indicate that sequencing a single affected patient can identify the causative mutations in known IRD genes when consanguinity is present. The identification of causative mutations enables appropriate molecular diagnosis that is critical for choosing therapeutic options as gene based therapies become available. The lack of identification of mutations in known IRD genes in half of the pedigrees analyzed suggests that novel genes are involved in causing IRD pathology in some of these families. Identification of additional genes involved in IRD pathology will further improve our understanding of the molecular pathology of retinal degenerations.

## Supporting Information

S1 Table Exome Capture and Variant Calling Statistics. (DOCX)

S2 Table List of IRD genes queried in this study. (DOCX)

S3 Table Sequence of primers used in this study. (DOCX)

S4 Table Details of variants detected in exomes sequenced. (DOCX)

S5 Table Candidate variants identified in Pakistani pedigrees. (DOCX)

S6 Table SNVs unique and common in Pakistani population. (DOCX)

## References

[1] RetNet. Available from: http://www.sph.uth.tmc.edu/Retnet/(as of Dec 8, 2014).

[2] Weleber RG, Carr RE, Murphey WH, Sheffield VC, Stone EM. Phenotypic variation including retinitis pigmentosa, pattern dystrophy, and fundus flavimaculatus in a single family with a deletion of codon 153 or 154 of the peripherin/RDS gene. Arch Ophthalmol. 1993;111(11):1531–42. . 8240110 10.1001/archopht.1993.01090110097033

[3] Ayyagari R, Mandal MN, Karoukis AJ, Chen L, McLaren NC, Lichter M, et al Late-onset macular degeneration and long anterior lens zonules result from a CTRP5 gene mutation. Invest Ophthalmol Vis Sci. 2005;46(9):3363–71. 10.1167/iovs.05-0159 . 16123441

[4] Li L, Nakaya N, Chavali VR, Ma Z, Jiao X, Sieving PA, et al A mutation in ZNF513, a putative regulator of photoreceptor development, causes autosomal-recessive retinitis pigmentosa. Am J Hum Genet. 2010;87(3):400–9. 10.1016/j.ajhg.2010.08.003 20797688 PMC2933346

[5] Branham K, Othman M, Brumm M, Karoukis AJ, Atmaca-Sonmez P, Yashar BM, et al Mutations in RPGR and RP2 account for 15% of males with simplex retinal degenerative disease. Investigative ophthalmology & visual science. 2012;53(13):8232–7. 10.1167/iovs.12-11025 23150612 PMC3522443

[6] Brown MD, Voljavec AS, Lott MT, MacDonald I, Wallace DC. Leber's hereditary optic neuropathy: a model for mitochondrial neurodegenerative diseases. FASEB journal: official publication of the Federation of American Societies for Experimental Biology. 1992;6(10):2791–9. . 1634041 10.1096/fasebj.6.10.1634041

[7] Hearn T, Renforth GL, Spalluto C, Hanley NA, Piper K, Brickwood S, et al Mutation of ALMS1, a large gene with a tandem repeat encoding 47 amino acids, causes Alstrom syndrome. Nature genetics. 2002;31(1):79–83. 10.1038/ng874 . 11941370

[8] Wang X, Wang H, Cao M, Li Z, Chen X, Patenia C, et al Whole-exome sequencing identifies ALMS1, IQCB1, CNGA3, and MYO7A mutations in patients with Leber congenital amaurosis. Human mutation. 2011;32(12):1450–9. 10.1002/humu.21587 21901789 PMC3943164

[9] Daiger SP, Sullivan LS, Bowne SJ. Genes and mutations causing retinitis pigmentosa. Clinical genetics. 2013;84(2):132–41. 10.1111/cge.12203 23701314 PMC3856531

[10] Abu-Safieh L, Alrashed M, Anazi S, Alkuraya H, Khan AO, Al-Owain M, et al Autozygome-guided exome sequencing in retinal dystrophy patients reveals pathogenetic mutations and novel candidate disease genes. Genome research. 2013;23(2):236–47. 10.1101/gr.144105.112 23105016 PMC3561865

[11] Jinda W, Taylor TD, Suzuki Y, Thongnoppakhun W, Limwongse C, Lertrit P, et al Whole exome sequencing in Thai patients with retinitis pigmentosa reveals novel mutations in six genes. Investigative ophthalmology & visual science. 2014;55(4):2259–68. 10.1167/iovs.13-13567 . 24618324

[12] Fu Q, Wang F, Wang H, Xu F, Zaneveld JE, Ren H, et al Next-generation sequencing-based molecular diagnosis of a Chinese patient cohort with autosomal recessive retinitis pigmentosa. Investigative ophthalmology & visual science. 2013;54(6):4158–66. 10.1167/iovs.13-11672 23661369 PMC3684217

[13] Chen Y, Zhang Q, Shen T, Xiao X, Li S, Guan L, et al Comprehensive mutation analysis by whole-exome sequencing in 41 Chinese families with Leber congenital amaurosis. Investigative ophthalmology & visual science. 2013;54(6):4351–7. 10.1167/iovs.13-11606 . 23661368

[14] Khan SJ, Hassan A, Khalid L, Karim U, Hashmi E, Gul F, et al Blindness in children at the Ida Rieu school for the blind and deaf. JPMA The Journal of the Pakistan Medical Association. 2007;57(7):334–7. . 17867253

[15] Khan MI, Azam M, Ajmal M, Collin RW, den Hollander AI, Cremers FP, et al The molecular basis of retinal dystrophies in pakistan. Genes. 2014;5(1):176–95. 10.3390/genes5010176 24705292 PMC3978518

[16] McKibbin M, Ali M, Mohamed MD, Booth AP, Bishop F, Pal B, et al Genotype-phenotype correlation for leber congenital amaurosis in Northern Pakistan. Archives of ophthalmology. 2010;128(1):107–13. 10.1001/archophthalmol.2010.309 . 20065226

[17] Ali S, Khan SY, Naeem MA, Khan SN, Husnain T, Riazuddin S, et al Phenotypic variability associated with the D226N allele of IMPDH1. Ophthalmology. 2015;122(2):429–31. 10.1016/j.ophtha.2014.07.057 . 25439607

[18] Ali S, Riazuddin SA, Shahzadi A, Nasir IA, Khan SN, Husnain T, et al Mutations in the beta-subunit of rod phosphodiesterase identified in consanguineous Pakistani families with autosomal recessive retinitis pigmentosa. Molecular vision. 2011;17:1373–80. 21655355 PMC3108895

[19] Chassine T, Bocquet B, Daien V, Avila-Fernandez A, Ayuso C, Collin RW, et al Autosomal recessive retinitis pigmentosa with RP1 mutations is associated with myopia. Br J Ophthalmol. 2015 10.1136/bjophthalmol-2014-306224 . 25883087 PMC12018887

[20] Iqbal M, Naeem MA, Riazuddin SA, Ali S, Farooq T, Qazi ZA, et al Association of pathogenic mutations in TULP1 with retinitis pigmentosa in consanguineous Pakistani families. Archives of ophthalmology. 2011;129(10):1351–7. 10.1001/archophthalmol.2011.267 21987678 PMC3463811

[21] Li D, Jin C, Jiao X, Li L, Bushra T, Naeem MA, et al AIPL1 implicated in the pathogenesis of two cases of autosomal recessive retinal degeneration. Molecular vision. 2014;20:1–14. 24426771 PMC3888496

[22] Naeem MA, Chavali VR, Ali S, Iqbal M, Riazuddin S, Khan SN, et al GNAT1 associated with autosomal recessive congenital stationary night blindness. Invest Ophthalmol Vis Sci. 2012;53(3):1353–61. 10.1167/iovs.11-8026 22190596 PMC3339909

[23] Riazuddin SA, Shahzadi A, Zeitz C, Ahmed ZM, Ayyagari R, Chavali VR, et al A mutation in SLC24A1 implicated in autosomal-recessive congenital stationary night blindness. Am J Hum Genet. 2010;87(4):523–31. 10.1016/j.ajhg.2010.08.013 20850105 PMC2948789

[24] Kaul H, Riazuddin SA, Yasmeen A, Mohsin S, Khan M, Nasir IA, et al A new locus for autosomal recessive congenital cataract identified in a Pakistani family. Molecular vision. 2010;16:240–5. 20161816 PMC2822550

[25] Kaul H, Riazuddin SA, Shahid M, Kousar S, Butt NH, Zafar AU, et al Autosomal recessive congenital cataract linked to EPHA2 in a consanguineous Pakistani family. Molecular vision. 2010;16:511–7. 20361013 PMC2846848

[26] Duncan JL, Roorda A, Navani M, Vishweswaraiah S, Syed R, Soudry S, et al Identification of a novel mutation in the CDHR1 gene in a family with recessive retinal degeneration. Arch Ophthalmol. 2012;130(10):1301–8. 10.1001/archophthalmol.2012.1906 23044944 PMC3799916

[27] Maranhao B, Biswas P, Duncan JL, Branham KE, Silva GA, Naeem MA, et al exomeSuite: Whole exome sequence variant filtering tool for rapid identification of putative disease causing SNVs/indels. Genomics. 2014;103(2–3):169–76. 10.1016/j.ygeno.2014.02.006 . 24603341 PMC4146529

[28] International HapMap C, Altshuler DM, Gibbs RA, Peltonen L, Altshuler DM, Gibbs RA, et al Integrating common and rare genetic variation in diverse human populations. Nature. 2010;467(7311):52–8. 10.1038/nature09298 20811451 PMC3173859

[29] Genomes Project C, Abecasis GR, Altshuler D, Auton A, Brooks LD, Durbin RM, et al A map of human genome variation from population-scale sequencing. Nature. 2010;467(7319):1061–73. 10.1038/nature09534 20981092 PMC3042601

[30] exomevariantserver. W.NHLBIGO Exome Sequencing Project (ESP). http://evsgswashingedu/EVS/2013. 2013.

[31] Friedman RC, Farh KK, Burge CB, Bartel DP. Most mammalian mRNAs are conserved targets of microRNAs. Genome research. 2009;19(1):92–105. 10.1101/gr.082701.108 18955434 PMC2612969

[32] Schwarz JM, Rodelsperger C, Schuelke M, Seelow D. MutationTaster evaluates disease-causing potential of sequence alterations. Nature methods. 2010;7(8):575–6. 10.1038/nmeth0810-575 . 20676075

[33] Adzhubei IA, Schmidt S, Peshkin L, Ramensky VE, Gerasimova A, Bork P, et al A method and server for predicting damaging missense mutations. Nature methods. 2010;7(4):248–9. 10.1038/nmeth0410-248 20354512 PMC2855889

[34] Choi Y, Sims GE, Murphy S, Miller JR, Chan AP. Predicting the functional effect of amino acid substitutions and indels. PloS one. 2012;7(10):e46688 10.1371/journal.pone.0046688 23056405 PMC3466303

[35] Kumar P, Henikoff S, Ng PC. Predicting the effects of coding non-synonymous variants on protein function using the SIFT algorithm. Nature protocols. 2009;4(7):1073–81. 10.1038/nprot.2009.86 . 19561590

[36] Reese MG, Eeckman FH, Kulp D, Haussler D. Improved splice site detection in Genie. Journal of computational biology: a journal of computational molecular cell biology. 1997;4(3):311–23. . 9278062 10.1089/cmb.1997.4.311

[37] Simonelli F, Ziviello C, Testa F, Rossi S, Fazzi E, Bianchi PE, et al Clinical and molecular genetics of Leber's congenital amaurosis: a multicenter study of Italian patients. Investigative ophthalmology & visual science. 2007;48(9):4284–90. 10.1167/iovs.07-0068 . 17724218

[38] Gu SM, Thompson DA, Srikumari CR, Lorenz B, Finckh U, Nicoletti A, et al Mutations in RPE65 cause autosomal recessive childhood-onset severe retinal dystrophy. Nature genetics. 1997;17(2):194–7. 10.1038/ng1097-194 . 9326941

[39] Di Gioia SA, Letteboer SJ, Kostic C, Bandah-Rozenfeld D, Hetterschijt L, Sharon D, et al FAM161A, associated with retinitis pigmentosa, is a component of the cilia-basal body complex and interacts with proteins involved in ciliopathies. Human molecular genetics. 2012;21(23):5174–84. Epub 2012/09/04. 10.1093/hmg/dds368 . 22940612

[40] Langmann T, Di Gioia SA, Rau I, Stohr H, Maksimovic NS, Corbo JC, et al Nonsense mutations in FAM161A cause RP28-associated recessive retinitis pigmentosa. American journal of human genetics. 2010;87(3):376–81. 10.1016/j.ajhg.2010.07.018 20705278 PMC2933350

[41] Bandah-Rozenfeld D, Mizrahi-Meissonnier L, Farhy C, Obolensky A, Chowers I, Pe'er J, et al Homozygosity mapping reveals null mutations in FAM161A as a cause of autosomal-recessive retinitis pigmentosa. American journal of human genetics. 2010;87(3):382–91. 10.1016/j.ajhg.2010.07.022 20705279 PMC2933343

[42] Farkas MH, Grant GR, White JA, Sousa ME, Consugar MB, Pierce EA. Transcriptome analyses of the human retina identify unprecedented transcript diversity and 3.5 Mb of novel transcribed sequence via significant alternative splicing and novel genes. BMC genomics. 2013;14:486 10.1186/1471-2164-14-486 23865674 PMC3924432

[43] Huang XF, Huang F, Wu KC, Wu J, Chen J, Pang CP, et al Genotype-phenotype correlation and mutation spectrum in a large cohort of patients with inherited retinal dystrophy revealed by next-generation sequencing. Genetics in medicine: official journal of the American College of Medical Genetics. 2015;17(4):271–8. 10.1038/gim.2014.138 . 25356976

