# Supplementary material for: Investigating the Molecular Basis of Retinal Degeneration in a Familial Cohort of Pakistani Decent by Exome Sequencing
Source: PLoS One. 2015 Sep 9;10(9):e0136561. doi: 10.1371/journal.pone.0136561 (PMC4564165; doi:10.1371/journal.pone.0136561)
Supplement: S1 Table — (DOCX) [file pone.0136561.s001.docx]

S1 Table – Exome capture and variant calling statistics.

| **Pedigree** | **Member** | **Capture Kit** | **Bases Sequenced (Gbp)** | **Average Depth in Targeted Regions** | **# of Variants Called** | **Proportion of Variants Called Homozygous** |
| --- | --- | --- | --- | --- | --- | --- |
| **PKRP077** | IV:5 | Nimblegen V2 | 3.48 | 29.72 | 24,139 | 48.56% |
| **PKRP078** | IV:6 | Nimblegen V2 | 9.83 | 79.95 | 46,807 | 46.02% |
| **PKRP078** | IV:9 | Nimblegen V2 | 3.68 | 29.55 | 29,389 | 45.96% |
| **PKRP103** | IV:2 | Nimblegen V2 | 4.9 | 36.44 | 31,600 | 42.50% |
| **PKRP138** | V:3 | Agilent V5+UTRs | 6.61 | 40.33 | 71,806 | 40.81% |
| **PKRP141** | V:2 | Agilent V5+UTRs | 5.14 | 30.6 | 68,665 | 43.45% |
| **PKRP142** | V:13 | Agilent V5+UTRs | 6.64 | 39.57 | 70,968 | 40.37% |
| **PKRP176** | V:3 | Agilent V5+UTRs | 7.01 | 41.35 | 70,264 | 42.15% |
| **PKRP185** | IV:2 | Agilent V5+UTRs | 7.18 | 42.2 | 70,399 | 40.53% |
| **PKRP281** | V:5 | Nimblegen V3 | 8.92 | 68.04 | 50,542 | 41.40% |
| **PKRP282** | IV:7 | Agilent V4 | 9.55 | 107.03 | 45,357 | 40.87% |
| **PKRP282** | IV:12 | Agilent V4 | 9.55 | 107.99 | 46,549 | 40.50% |
| **PKRP282** | IV:13 | Agilent V4 | 8.56 | 94.84 | 44,847 | 42.39% |
| **PKRP283** | IV:1 | Agilent V4 | 8.34 | 92.76 | 44,427 | 43.67% |
| **PKRP283** | IV:12 | Agilent V4 | 9.69 | 109.18 | 46,100 | 38.47% |
| **PKRP284** | IV:14 | Nimblegen V3 | 8.23 | 50.16 | 51,221 | 41.79% |
| **PKRP284** | IV:17 | Nimblegen V3 | 15.74 | 108.74 | 51,466 | 43.94% |
