# Supplementary material for: Investigating the Molecular Basis of Retinal Degeneration in a Familial Cohort of Pakistani Decent by Exome Sequencing
Source: PLoS One. 2015 Sep 9;10(9):e0136561. doi: 10.1371/journal.pone.0136561 (PMC4564165; doi:10.1371/journal.pone.0136561)
Supplement: S2 Table — (DOCX) [file pone.0136561.s002.docx]

S2 Table – List of IRD genes queried in this study.

| AA | ABCA4 | ABCC6 | ABCR | ABHD12 | ABL |
| --- | --- | --- | --- | --- | --- |
| ABRI | ACBD5 | ACDP4 | ACHM1 | ACHM2 | ACHM3 |
| ACHM4 | ADAM9 | ADAMTS18 | ADCA2 | ADNIV | AGS |
| AGS1 | AHI1 | AIED | AIPL1 | ALMS1 | ALS2CR4 |
| ALSS | AOM | ARA | ARL2BP | ARL6 | ARMD1 |
| ARMD10 | ARMD2 | ARMD3 | ARMD4 | ARMD5 | ARMD6 |
| ARMD7 | ARMD8 | ARMD9 | ARMS1 | ARMS2 | ASCC3L1 |
| ASP | ATD5 | ATP6 | ATXN7 | AXPC1 | BART |
| BART1 | BBIP1 | BBIP10 | BBS1 | BBS10 | BBS11 |
| BBS12 | BBS13 | BBS14 | BBS15 | BBS16 | BBS17 |
| BBS18 | BBS2 | BBS2L1 | BBS3 | BBS4 | BBS5 |
| BBS6 | BBS7 | BBS8 | BBS9 | BCAMD | BCD |
| BCM | BCP | BEST1 | BF | BFD | BRR2 |
| BSMD | C12orf65 | C1QTNF5 | C1orf36 | C2 | C21orf2 |
| C2orf71 | C3 | C8orf37 | CA4 | CABP4 | CACD |
| CACD1 | CACD2 | CACNA1F | CACNA2D4 | CAPN5 | CBD |
| CBP | CBT | CC2D2A | CCCAP | CDH23 | CDH3 |
| CDHP | CDHR1 | CDMP2 | CED4 | CEP164 | CEP290 |
| CERKL | CFB | CFH | CHBL | CHM | CIB2 |
| CLN3 | CLRN1 | CMT2A2 | CMT6 | CNCG | CNCG1 |
| CNCG2 | CNCG3 | CNCG3L | CNGA1 | CNGA3 | CNGB1 |
| CNGB3 | CNNM4 | COD1 | COD2 | COD3 | COD4 |
| COD5 | CODA1 | COL11A1 | COL2A1 | COL9A1 | CORD1 |
| CORD10 | CORD11 | CORD12 | CORD13 | CORD14 | CORD15 |
| CORD16 | CORD17 | CORD2 | CORD3 | CORD4 | CORD5 |
| CORD6 | CORD7 | CORD8 | CORD9 | CORDX1 | CORDX2 |
| CORDX3 | CORS1 | CORS2 | COXPD7 | CRALBP | CRB1 |
| CRV | CRX | CSNB1 | CSNB1A | CSNB1B | CSNB1C |
| CSNB1D | CSNB1E | CSNB2 | CSNB2A | CSNB2B | CSNB3 |
| CSNB4 | CSNBAD1 | CSNBAD2 | CSNBAD3 | CSNBX2 | CSPG2 |
| CSPP1 | CTRP5 | CYMD | CYP4V2 | DDP | DDP2 |
| DFN1 | DFNA38 | DFNB12 | DFNB18 | DFNB2 | DFNB23 |
| DFNB31 | DFNB48 | DHDDS | DHRD | DHX38 | DMD |
| DMDF | DTHD1 | EAR3 | EFEMP1 | EG5 | ELOVL4 |
| EMC1 | ERCC6 | ERVR | ESCS | EVR1 | EVR2 |
| EVR3 | EVR4 | EYS | FAM161A | FBLN3 | FBLN5 |
| FBLN6 | FEB4 | FEVR | FHASD | FIGLER4 | FLJ23560 |
| FLJ35630 | FLVCR1 | FRITZ | FSCN2 | FZD4 | GAR1 |
| GARP | GCAP1 | GCAP2 | GCP | GDF6 | GNAT1 |
| GNAT2 | GNPTG | GPR125 | GPR179 | GPR98 | GRK1 |
| GRM6 | GUCA1A | GUCA1B | GUCY2D | HARP | HARS |
| HBM | HECIC2 | HF1 | HK1 | HK5P | HMCN1 |
| HPRP3 | HRG4 | HRS | HSD3 | HT2A | HTRA1 |
| HTRA3 | IDH3B | IFT140 | IFT144 | IFT27 | IMPDH1 |
| IMPG1 | IMPG2 | INPP5E | INVS | IQCB1 | IRBP |
| IRD | ITM2B | JAG1 | JBTS1 | JBTS14 | JBTS19 |
| JBTS2 | JBTS3 | JBTS4 | JBTS5 | JBTS7 | JBTS9 |
| JNCL | KCNJ13 | KCNV2 | KFS1 | KIAA1005 | KIAA1549 |
| KIF11 | KIP2 | KIZ | KLHL7 | KNO2 | KNSL1 |
| KSS | LCA1 | LCA10 | LCA11 | LCA12 | LCA13 |
| LCA14 | LCA15 | LCA16 | LCA17 | LCA2 | LCA3 |
| LCA4 | LCA5 | LCA6 | LCA7 | LCA8 | LCA9 |
| LHON | LOC387715 | LOC619531 | LRAT | LRIT3 | LRP5 |
| LUN | LZTFL1 | MAK | MARF | MASS1 | MCDCA |
| MCDR1 | MCDR2 | MCDR3 | MCDR4 | MCDR5 | MCLMR |
| MCMP | MDC9 | MDDC | MERTK | MFN2 | MFRP |
| MGA3 | MKKS | MKS1 | MKS4 | MKS5 | MKS6 |
| MLSN1 | MRP6 | MRST | MT-ATP6 | MT-TH | MT-TL1 |
| MT-TP | MT-TS2 | MTLV | MTP | MTTP | MVK |
| MYO7A | MZSDS | NARP | NCKX | NCMD | NDP |
| NEK2 | NET2 | NIR1 | NLK1 | NMNAT1 | NNO2 |
| NPHP1 | NPHP10 | NPHP13 | NPHP14 | NPHP15 | NPHP2 |
| NPHP3 | NPHP4 | NPHP5 | NPHP6 | NPHP8 | NR2E3 |
| NR2F1 | NRL | NYX | OA2 | OAT | OFD1 |
| ONCR | OPA1 | OPA2 | OPA3 | OPA4 | OPA5 |
| OPA6 | OPA7 | OPA8 | OPCA3 | OPN1LW | OPN1MW |
| OPN1SW | OPN2 | OPPG | ORP1 | OTX2 | P53BP3 |
| PAF1 | PAHX | PANK2 | PAP1 | PAX2 | PBCRA1 |
| PCAD | PCDH15 | PCDH21 | PCYT1A | PDE6A | PDE6B |
| PDE6C | PDE6G | PDE6H | PDEA2 | PDZD7 | PDZK7 |
| PEX1 | PEX2 | PEX7 | PGK1 | PGR21 | PHARC |
| PHYH | PIM1K | PITPNM3 | PKAN | PLA2G5 | PMP35 |
| PNAT1 | PNR | PRCD | PRD | PROM1 | PROML1 |
| PRP16 | PRP3 | PRP31 | PRPC8 | PRPF3 | PRPF31 |
| PRPF4 | PRPF6 | PRPF8 | PRPH2 | PRSS11 | PTHB1 |
| PTS2R | PWDMP | PXE | PXMP3 | QRX | R9AP |
| RAB28 | RAX2 | RAXL1 | RB1 | RBP3 | RBP4 |
| RCD1 | RCD2 | RCD3A | RCD3B | RCD4 | RCDP1 |
| RCP | RD3 | RDH1 | RDH11 | RDH12 | RDH5 |
| RDPA | RDS | RETGC | RETGC1 | RGR | RGS9 |
| RGS9BP | RHO | RHOK | RIM1 | RIMS1 | RK |
| RLBP1 | RMCH | RMCH1 | RMCH2 | RNANC | ROA1 |
| RODX | ROM1 | RP1 | RP10 | RP11 | RP12 |
| RP13 | RP14 | RP15 | RP16 | RP17 | RP18 |
| RP19 | RP1L1 | RP2 | RP20 | RP21 | RP22 |
| RP23 | RP24 | RP25 | RP26 | RP27 | RP28 |
| RP29 | RP3 | RP30 | RP31 | RP32 | RP33 |
| RP34 | RP35 | RP36 | RP37 | RP38 | RP39 |
| RP4 | RP40 | RP41 | RP42 | RP43 | RP44 |
| RP45 | RP46 | RP47 | RP48 | RP49 | RP5 |
| RP50 | RP51 | RP53 | RP54 | RP55 | RP56 |
| RP57 | RP58 | RP59 | RP6 | RP60 | RP61 |
| RP62 | RP63 | RP64 | RP65 | RP66 | RP67 |
| RP68 | RP69 | RP7 | RP8 | RP9 | RPE65 |
| RPGR | RPGRIP1 | RPGRIP1L | RS1 | RVCL | SAG |
| SANS | SCA7 | SDCCAG8 | SEMA4A | SEMAB | SFD |
| SLC24A1 | SLC7A14 | SLSN1 | SLSN3 | SLSN4 | SLSN5 |
| SLSN6 | SLSN7 | SNRNP200 | SPAM | SPARC | SPARCAN |
| SPATA7 | SPG55 | STAMP | STGD1 | STGD2 | STGD3 |
| STGD4 | STL1 | STL2 | SVD | TCF13 | TEAD1 |
| TEF1 | TEM5L | TIMM8A | TIMP3 | TLR3 | TLR4 |
| TMEM126A | TMEM237 | TOPORS | TREX1 | TRIM32 | TRIP5 |
| TRNH | TRNL1 | TRNP | TRNS2 | TRPM1 | TSPAN12 |
| TTC8 | TTLL5 | TTPA | TU15B | TUB | TULP1 |
| UNC119 | USH1 | USH1A | USH1B | USH1C | USH1D |
| USH1E | USH1F | USH1G | USH1H | USH1J | USH1K |
| USH2A | USH2B | USH2C | USH2D | USH3 | USH3A |
| USH3B | VCAN | VLGR1 | VMD1 | VMD2 | VRD1 |
| VRNI | WDPCP | WDR19 | WDTC2 | WFS1 | WFS2 |
| WGN1 | WHRN | XLRS1 | ZNF423 | ZNF513 |  |
