# Supplementary material for: Investigating the Molecular Basis of Retinal Degeneration in a Familial Cohort of Pakistani Decent by Exome Sequencing
Source: PLoS One. 2015 Sep 9;10(9):e0136561. doi: 10.1371/journal.pone.0136561 (PMC4564165; doi:10.1371/journal.pone.0136561)
Supplement: S3 Table — (DOCX) [file pone.0136561.s003.docx]

S3 Table – Sequence of primers used in this study.

| **Target** | **Forward Primer** | **Reverse Primer** | **Product Size (bp)** |
| --- | --- | --- | --- |
| CNGB1 Exon 26 | ACCCATGTTCCCTTTCTCCT | AGGGTGACATTTCTGGCAAC | 249 |
| FAM161A Exon 4 | ATCCCATGTTAAATCTTTGC | GAAAACCAGTGGTCTGGAG | 284 |
| GUCY2D Exon 12 | AGGCCAGGGTCAGAGGCAGC | CTCAGGTTGCTGACAAGCATC | 286 |
| LCA5 Exon 7 | TCTGTGTTGCTTAGTTCCCC | TTTCTTTCTCAAGGGATGCTG | 256 |
| RPE65 Exon 10 | TTGTCATTGCCTGTGCTCATG | TGAGAGAGATGAAACATTCTGG | 226 |
| USH2A Exon 12 | CCCTGTCTTGTACCTAATGAGC | TGACTGTAAACTTTTGCGTTACAC | 302 |
| USH2A Exon 22 | AGCAGGAATGTGGGCATAAG | GATTCAGTGTGAAAACAGAAGCA | 434 |
| USH2A Exon 59 | TGTTTTTAATGTTCTCTCTCTCTCTCT | CCAGGCCAAGTGTCTGAAAG | 224 |
| USH2A Exon 63 | TTTGTCTCTCTGTGGGCCTT | CAAAGATCTGGAGGGCTGAC | 987 |
