# Supplementary material for: Investigating the Molecular Basis of Retinal Degeneration in a Familial Cohort of Pakistani Decent by Exome Sequencing
Source: PLoS One. 2015 Sep 9;10(9):e0136561. doi: 10.1371/journal.pone.0136561 (PMC4564165; doi:10.1371/journal.pone.0136561)
Supplement: S4 Table — (DOCX) [file pone.0136561.s004.docx]

S4 Table – Details of variants detected in exomes sequenced.

| **Pedigree** | **# of Affected Individuals Sequenced** | **# of Unaffected Individuals Sequenced** | **Total # of Variants Identified** | **Variants Segregating with Disease** | **Variants Satisfying Filter Criteria** | **Variants Located in Known RD Genes** |
| --- | --- | --- | --- | --- | --- | --- |
| **PKRP077** | 1 | 0 | 24,139 | 11,721 | 328 | 5 |
| **PKRP078** | 1 | 1 | 57,017 | 5,187 | 279 | 6 |
| **PKRP103** | 1 | 0 | 31,600 | 13,430 | 243 | 4 |
| **PKRP138** | 1 | 0 | 71,806 | 29,304 | 1,064 | 12 |
| **PKRP141** | 1 | 0 | 68,665 | 29,835 | 1,209 | 19 |
| **PKRP142** | 1 | 0 | 70,968 | 28,650 | 1,060 | 9 |
| **PKRP176** | 1 | 0 | 70,264 | 29,616 | 1,184 | 25 |
| **PKRP185** | 1 | 0 | 70,399 | 28,533 | 958 | 7 |
| **PKRP281** | 1 | 0 | 50,542 | 20,924 | 536 | 16 |
| **PKRP282** | 1 | 1 | 66,059 | 10,356 | 798 | 19 |
| **PKRP283** | 2 | 0 | 58,480 | 25,153 | 855 | 7 |
| **PKRP284** | 1 | 1 | 61.935 | 5,057 | 264 | 4 |
