# Supplementary material for: Investigating the Molecular Basis of Retinal Degeneration in a Familial Cohort of Pakistani Decent by Exome Sequencing
Source: PLoS One. 2015 Sep 9;10(9):e0136561. doi: 10.1371/journal.pone.0136561 (PMC4564165; doi:10.1371/journal.pone.0136561)
Supplement: S5 Table — (DOCX) [file pone.0136561.s005.docx]

S5 Table – Candidate variants identified in Pakistani pedigrees.

| Pedigree | CHR | POS | rsID | REF | ALT | GENE | Transcript ID | Peptide ID | cDNA POS | AA Change | HapMap | 1000 | ESP6500 | ExAC | PolyPhen2 | Mutation | SIFT | PROVEAN |
| --- | --- | --- | --- | --- | --- | --- | --- | --- | --- | --- | --- | --- | --- | --- | --- | --- | --- | --- |
|  |  |  |  |  |  |  |  |  |  |  |  | Genome | AF |  |  |  |  |  |
| PKRD077 | 1 | 103468336 |  | A | C | COL11A1 | ENST00000370096 | ENSP00000359114 | c.2010T>G | p.(=) | 0 | 0.0002 | 0 | 0 | silent mutation | disease_causing | Tolerated | Neutral |
| PKRD077 | 17 | 7917318 | rs61750171 | G | A | GUCY2D | ENST00000254854 | ENSP00000254854 | c.2384G>A | p.R795Q | 0 | 0.0026 | 0 | 0.0024 | probably damaging | disease_causing | Damaging | Deleterious |
| PKRD078 | 2 | 29294239 | rs144569618 | G | A | C2orf71 | ENST00000331664 | ENSP00000332809 | c.2889C>T | p.(=) | 0 | 0.0008 | 0 | 0.0006 | silent mutation | polymorphism | Tolerated | Neutral |
| PKRD078 | 2 | 182521668 | rs199762900 | G | C | CERKL | ENST00000339098 | ENSP00000341159 | c.66C>G | p.(=) | 0 | 0.0002 | 0 | 0.0002 | silent mutation | polymorphism | Tolerated | Neutral |
| PKRD078 | 6 | 65707465 | rs78511049 | G | A | EYS | ENST00000370621 | ENSP00000359655 | c.2259+10C>T |  | 0 | 0 | 0 | 0.0174 |  |  |  |  |
| PKRD078 | 6 | 80198880 | rs386834252 | TG | T | LCA5 | ENST00000369846 | ENSP00000358861 | c.1149C[2] | p.(N383fs) | 0 | 0.013 | 0.0002 | 0 |  |  |  |  |
| PKRD078 | 12 | 110017759 | rs67886029 | C | T | MVK | ENST00000228510 | ENSP00000228510 | c.371+8C>T |  | 0 | 0 | 0 | 0 |  |  |  |  |
| PKRD103 | 17 | 7916496 |  | T | C | GUCY2D | ENST00000254854 | ENSP00000254854 | c.2189T>C | p.F730S | 0 | 0 | 0 | 0 | probably damaging | disease_causing | Damaging | Deleterious |
| PKRD138 | 1 | 103469989 | rs71664954 | A | G | COL11A1 | ENST00000370096 | ENSP00000359114 | c.1944+11T>C |  | 0 | 0 | 0 | 0.0023 |  |  |  |  |
| PKRD138 | 1 | 215916593 |  | TG | T | USH2A | ENST00000307340 | ENSP00000305941 | c.11473del | p.(H3825fs) | 0 | 0.0034 | 0 | 0.051 |  |  |  |  |
| PKRD138 | 1 | 216465626 | rs41313732 | G | A | USH2A | ENST00000307340 | ENSP00000305941 | c.1731C>T | p.(=) | 0.0213 | 0.0222 | 0.0366 | 0.0733 | silent mutation | disease_causing | Tolerated | Neutral |
| PKRD138 | 15 | 65918176 | rs370680044 | CCTG | C | SLC24A1 | ENST00000261892 | ENSP00000261892 | c.1759_1761CTG[4] | p.(L587[4]) | 0 | 0.0689 | 0.0365 | 0 |  |  |  | Deleterious |
| PKRD141 | 1 | 211652533 |  | G | T | RD3 | ENST00000367002 | ENSP00000355969 | c.433C>A | p.(=) | 0 | 0.0002 | 0 | 0 | silent mutation | disease_causing | Tolerated | Neutral |
| PKRD141 | 1 | 216270538 | rs199679165 | G | A | USH2A | ENST00000307340 | ENSP00000305941 | c.4645C>T | p.R1549* | 0 | 0.0026 | 0 | 0.0024 |  |  |  |  |
| PKRD141 | 11 | 61730439 | rs566374710 | C | A | BEST1 | ENST00000449131 | ENSP00000399709 | c.1633C>A | p.P545T | 0 | 0.0008 | 0 | 0.0006 |  | polymorphism | Damaging | Neutral |
| PKRD141 | 11 | 68207342 | rs142191419 | G | C | LRP5 | ENST00000294304 | ENSP00000294304 | c.4446G>C | p.(=) | 0 | 0.0002 | 0 | 0.0002 | silent mutation | disease_causing | Tolerated | Neutral |
| PKRD142 | 1 | 243481345 | rs555521073 | T | G | SDCCAG8 | ENST00000391846 | ENSP00000375721 | c.1075T>G | p.S359A | 0 | 0 | 0 | 0.0174 |  | polymorphism | Damaging | Neutral |
| PKRD142 | 11 | 117253636 | rs74388237 | A | C | CEP164 | ENST00000278935 | ENSP00000278935 | c.1702A>C | p.T568P | 0 | 0.013 | 0.0002 | 0 | possibly damaging | polymorphism | Tolerated | Neutral |
| PKRD142 | 16 | 57938758 | rs370767664 | A | G | CNGB1 | ENST00000251102 | ENSP00000251102 | c.2514T>C | p.(=) | 0 | 0 | 0 | 0 | silent mutation | disease_causing | Tolerated | Neutral |
| PKRD142 | 16 | 57938776 |  | ATAACT | AGCC | CNGB1 | ENST00000251102 | ENSP00000251102 | c.2493-2_c.2495 |  | 0 | 0 | 0 | 0 |  |  |  |  |
|  |  |  |  |  |  |  |  |  | delins GGC |  |  |  |  |  |  |  |  |  |
| PKRD176 | 2 | 62065824 |  | T | A | FAM161A | ENST00000404929 | ENSP00000385158 | c.1600A>T | p.K534* | 0 | 0 | 0 | 0.0000088 |  |  |  |  |
|  |  |  |  |  |  |  |  |  |  |  |  |  |  |  |  |  |  |  |
| PKRD176 | 2 | 73678899 | rs546111188 | A | G | ALMS1 | ENST00000264448 | ENSP00000264448 | c.5242A>G | p.T1748A | 0 | 0.0034 | 0 | 0.0023 | probably damaging | polymorphism | Damaging | Deleterious |
| PKRD176 | 4 | 110772711 | rs17040904 | C | T | LRIT3 | ENST00000594814 | ENSP00000469759 | c.168C>T | p.(=) | 0.0213 | 0.0222 | 0.0366 | 0.0733 | silent mutation | disease_causing | Tolerated | Neutral |
| PKRD176 | 11 | 17531146 | rs17776775 | G | A | USH1C | ENST00000005226 | ENSP00000005226 | c.1770C>T | p.(=) | 0 | 0.0689 | 0.0365 | 0 | silent mutation | disease_causing | Tolerated | Neutral |
| PKRD176 | 13 | 48921964 |  | A | T | RB1 | ENST00000267163 | ENSP00000267163 | c.504A>T | p.(=) | 0 | 0.0002 | 0 | 0 | silent mutation | disease_causing | Tolerated | Neutral |
| PKRD176 | 17 | 6386947 | rs34897053 | G | A | PITPNM3 | ENST00000262483 | ENSP00000262483 | c.477C>T | p.(=) | 0 | 0 | 0 | 0.0472 | silent mutation | disease_causing | Tolerated | Neutral |
| PKRD176 | 19 | 3770938 |  | C | T | RAX2 | ENST00000555978 | ENSP00000450687 | c.236G>A | p.R79Q | 0 | 0.0665 | 0.0175 | 0.0001 | probably damaging | disease_causing | Damaging | Deleterious |
| PKRD185 | 1 | 215848730 |  | A | C | USH2A | ENST00000307340 | ENSP00000305941 | c.12523T>G | p.W4175G | 0 | 0 | 0 | 0 | probably damaging | disease_causing | Damaging | Deleterious |
| PKRD185 | 20 | 25282967 | rs746748 | C | T | ABHD12 | ENST00000339157 | ENSP00000341408 | c.1045G>A | p.A349T | 0.0263 | 0.0184 | 0.0427 | 0.0429 | possibly damaging | disease_causing | Tolerated | Neutral |
| PKRD281 | 1 | 68903911 | rs121917744 | G | T | RPE65 | ENST00000262340 | ENSP00000262340 | c.1087C>A | p.P363T | 0 | 0 | 0 | 0 | possibly damaging | disease_causing | Tolerated | Neutral |
| PKRD281 | 6 | 66005740 | rs74667330 | G | GA | EYS | ENST00000370621 | ENSP00000359655 | c.2023+6T[11] |  | 0 | 0 | 0 | 0.4891 |  |  |  |  |
| PKRD281 | 20 | 10622501 | rs35761929 | G | C | JAG1 | ENST00000254958 | ENSP00000254958 | c.2612C>G | p.P871R | 0 | 0.0529 | 0.0481 | 0.0662 | probably damaging | disease_causing | Damaging | Deleterious |
| PKRD282 | 1 | 68903911 | rs121917744 | G | T | RPE65 | ENST00000262340 | ENSP00000262340 | c.1087C>A | p.P363T | 0 | 0 | 0 | 0 | possibly damaging | disease_causing | Tolerated | Neutral |
| PKRD283 | 1 | 68903911 | rs121917744 | G | T | RPE65 | ENST00000262340 | ENSP00000262340 | c.1087C>A | p.P363T | 0 | 0 | 0 | 0 | possibly damaging | disease_causing | Tolerated | Neutral |
| PKRD284 | 1 | 68903911 | rs121917744 | G | T | RPE65 | ENST00000262340 | ENSP00000262340 | c.1087C>A | p.P363T | 0 | 0 | 0 | 0 | possibly damaging | disease_causing | Tolerated | Neutral |
| PKRD284 | 10 | 73558128 | rs41281334 | G | A | CDH23 | ENST00000224721 | ENSP00000224721 | c.6862G>A | p.V2288I | 0 | 0.0367 | 0.034 | 0.0496 | benign | disease_causing | Tolerated | Neutral |
