# Supplementary material for: Investigating the Molecular Basis of Retinal Degeneration in a Familial Cohort of Pakistani Decent by Exome Sequencing
Source: PLoS One. 2015 Sep 9;10(9):e0136561. doi: 10.1371/journal.pone.0136561 (PMC4564165; doi:10.1371/journal.pone.0136561)
Supplement: S6 Table — (DOCX) [file pone.0136561.s006.docx]

S6 Table – SNVs unique and common in Pakistani population.

| **CHR** | **POS** | **REF** | **ALT** | **# of** | **Gene** | **Strand** | **Transcript ID** | | **Peptide ID** | **Transcript Biotype** | **cDNA Pos** | | **Codon Change** | **Amino Acid Change** |
| --- | --- | --- | --- | --- | --- | --- | --- | --- | --- | --- | --- | --- | --- | --- |
|  |  |  |  | **Pedigrees** |  |  |  |  |  |  |  |  |  |  |
| 1 | 907631 | A | C | 11 | PLEKHN1 | + | ENST00000379410 | | ENSP00000368720 | protein_coding | c.866-37A>C | | |  |
| 1 | 13447844 | T | C | 6 | PRAMEF13 | - | ENST00000376132 | | ENSP00000365302 | protein_coding | c.*206A>G | |  |  |
| 1 | 16341139 | C | G | 7 | HSPB7 | - | ENST00000311890 | | ENSP00000310111 | protein_coding | c.*936G>C | |  |  |
| 1 | 19615324 | T | G | 8 | AKR7A3 | - | ENST00000361640 | | ENSP00000355377 | protein_coding | c.-121A>C | |  |  |
| 1 | 26090003 | T | C | 6 | MAN1C1 | + | ENST00000374332 | | ENSP00000363452 | protein_coding | c.1048-362T>C | | |  |
| 1 | 47905770 | G | A | 6 | FOXD2 | + | ENST00000334793 | | ENSP00000335493 | protein_coding | c.*475G>A | |  |  |
| 1 | 59247318 | A | C | 9 | JUN | - | ENST00000371222 | | ENSP00000360266 | protein_coding | c.*429T>G | |  |  |
| 1 | 59249213 | G | C | 6 | JUN | - | ENST00000371222 | | ENSP00000360266 | protein_coding | c.-471C>G | |  |  |
| 1 | 120165038 | A | G | 7 | ZNF697 | - | ENST00000421812 | | ENSP00000396857 | protein_coding | c.*290T>C | |  |  |
| 1 | 153777495 | T | C | 12 | GATAD2B | - | ENST00000368655 | | ENSP00000357644 | protein_coding | c.*5158A>G | |  |  |
| 1 | 153777499 | A | C | 9 | GATAD2B | - | ENST00000368655 | | ENSP00000357644 | protein_coding | c.*5154T>G | |  |  |
| 1 | 153777503 | A | C | 9 | GATAD2B | - | ENST00000368655 | | ENSP00000357644 | protein_coding | c.*5150T>G | |  |  |
| 1 | 159172820 | T | G | 7 | DARC | + | ENST00000368122 | | ENSP00000357104 | protein_coding | c.-679-u1251T>G | | |  |
| 1 | 159172820 | T | G | 7 | CADM3 | + | ENST00000368124 | | ENSP00000357106 | protein_coding | c.*1090+d1018T>G | | |  |
| 1 | 159172828 | T | G | 8 | DARC | + | ENST00000368122 | | ENSP00000357104 | protein_coding | c.-679-u1243T>G | | |  |
| 1 | 159172828 | T | G | 8 | CADM3 | + | ENST00000368124 | | ENSP00000357106 | protein_coding | c.*1090+d1026T>G | | |  |
| 1 | 179198317 | G | C | 7 | ABL2 | - | ENST00000502732 | | ENSP00000427562 | protein_coding | c.157+59C>G | | |  |
| 1 | 180168256 | C | A | 8 | QSOX1 | + | ENST00000367602 | | ENSP00000356574 | protein_coding | c.*2084C>A | |  |  |
| 1 | 237996664 | G | C | 10 | RYR2 | + | ENST00000366574 | | ENSP00000355533 | protein_coding | c.*717G>C | |  |  |
| 1 | 237996670 | G | C | 9 | RYR2 | + | ENST00000366574 | | ENSP00000355533 | protein_coding | c.*723G>C | |  |  |
| 1 | 248617150 | T | C | 10 | OR2T2 | + | ENST00000342927 | | ENSP00000343062 | protein_coding | c.*57+d20T>C | | |  |
| 1 | 248801511 | A | G | 5 | OR2T35 | - | ENST00000317450 | | ENSP00000324369 | protein_coding | c.*972+d77T>C | | |  |
| 1 | 248801528 | G | A | 8 | OR2T35 | - | ENST00000317450 | | ENSP00000324369 | protein_coding | c.*972+d60C>T | | |  |
| 1 | 248801538 | G | A | 5 | OR2T35 | - | ENST00000317450 | | ENSP00000324369 | protein_coding | c.*972+d50C>T | | |  |
| 2 | 96607003 | G | A | 5 | ANKRD36C | - | ENST00000456556 | | ENSP00000403302 | protein_coding | c.1492-56C>T | | |  |
| 3 | 9958016 | T | G | 6 | IL17RC | + | ENST00000295981 | | ENSP00000295981 | protein_coding | c.-218-u766T>G | | |  |
| 3 | 9958016 | T | G | 6 | IL17RE | + | ENST00000421412 | | ENSP00000404916 | protein_coding | c.*37+d488T>G | | |  |
| 3 | 9958020 | T | G | 5 | IL17RC | + | ENST00000295981 | | ENSP00000295981 | protein_coding | c.-218-u762T>G | | |  |
| 3 | 9958020 | T | G | 5 | IL17RE | + | ENST00000421412 | | ENSP00000404916 | protein_coding | c.*37+d492T>G | | |  |
| 3 | 10357168 | T | G | 6 | SEC13 | - | ENST00000350697 | | ENSP00000312122 | protein_coding | c.49-48A>C | |  |  |
| 3 | 49200058 | G | C | 8 | CCDC71 | - | ENST00000321895 | | ENSP00000319006 | protein_coding | c.*180C>G | |  |  |
| 7 | 6866319 | A | G | 9 | CCZ1B | - | ENST00000316731 | | ENSP00000314544 | protein_coding | c.-491T>C | |  |  |
| 7 | 36339394 | G | C | 7 | EEPD1 | + | ENST00000242108 | | ENSP00000242108 | protein_coding | c.*579G>C | |  |  |
| 7 | 44605736 | A | C | 7 | DDX56 | - | ENST00000258772 | | ENSP00000258772 | protein_coding | c.1567-58T>G | | |  |
| 7 | 102178357 | A | G | 5 | POLR2J3 | - | ENST00000379340 | | ENSP00000368645 | nonsense_mediated_decay | c.*1232+d9T>C | | |  |
| 7 | 142460482 | G | T | 12 | PRSS1 | + | ENST00000311737 | | ENSP00000308720 | protein_coding | c.591+64G>T | | |  |
| 7 | 142460495 | A | C | 15 | PRSS1 | + | ENST00000311737 | | ENSP00000308720 | protein_coding | c.591+77A>C | | |  |
| 7 | 142460503 | T | C | 9 | PRSS1 | + | ENST00000311737 | | ENSP00000308720 | protein_coding | c.591+85T>C | | |  |
| 7 | 142498706 | T | A | 7 | TRBC2 | + | ENST00000466254 | | ENSP00000417300 | TR_C_gene, 5' & 3' incomplete | n.-u19T>A | |  |  |
| 8 | 65289926 | T | C | 10 | MIR124-2 | + | ENST00000385081 | | | miRNA | n.-u1780T>C | | |  |
| 9 | 68409602 | G | C | 6 | RNA5SP284 | + | ENST00000384547 | | | rRNA | n.+d561G>C | |  |  |
| 9 | 97079430 | C | T | 5 | NUTM2F | - | ENST00000253262 | ENSP00000253262 | | protein_coding | c.*269+d1048G>A | | |  |
| 9 | 127253300 | A | C | 5 | NR5A1 | - | ENST00000373588 | ENSP00000362690 | | protein_coding | c.1138+60T>G | | |  |
| 10 | 23633741 | A | C | 5 | C10orf67 | - | ENST00000323327 | ENSP00000321464 | | protein_coding | c.-35T>G | |  |  |
| 10 | 50036968 | T | G | 6 | WDFY4 | + | ENST00000265453 | ENSP00000265453 | | protein_coding, 5' & 3' incomplete | n.600+27T>G | | |  |
| 10 | 51623602 | C | G | 11 | TIMM23 | - | ENST00000260867 | ENSP00000260867 | | protein_coding | c.-124-u264G>C | | |  |
| 10 | 115380483 | G | A | 5 | NRAP | - | ENST00000359988 | ENSP00000353078 | | protein_coding | c.2770-16C>T | | |  |
| 10 | 119302459 | G | A | 7 | EMX2 | + | ENST00000553456 | ENSP00000450962 | | protein_coding | c.-320G>A | |  |  |
| 11 | 1018091 | T | G | 8 | MUC6 | - | ENST00000421673 | ENSP00000406861 | | protein_coding | c.4710A>C | | ccA/ccC | p.(=) |
| 11 | 1018093 | G | T | 9 | MUC6 | - | ENST00000421673 | ENSP00000406861 | | protein_coding | c.4708C>A | | Cca/Aca | p.P1570T |
| 11 | 13032651 | T | G | 8 | RASSF10 | + | ENST00000529419 | | | processed_pseudogene | n.+d27T>G | |  |  |
| 11 | 27494130 | C | G | 6 | LGR4 | - | ENST00000379214 | ENSP00000368516 | | protein_coding | c.-281G>C | |  |  |
| 11 | 56854835 | C | G | 7 | . |  |  |  | |  |  | |  |  |
| 11 | 58346588 | T | G | 6 | ZFP91 | + | ENST00000316059 | ENSP00000339030 | | protein_coding | c.-167T>G | |  |  |
| 11 | 61919655 | T | G | 8 | INCENP | + | ENST00000278849 | ENSP00000278849 | | protein_coding | c.*207T>G | |  |  |
| 11 | 61919659 | T | G | 9 | INCENP | + | ENST00000278849 | ENSP00000278849 | | protein_coding | c.*211T>G | |  |  |
| 11 | 64374075 | A | C | 5 | NRXN2 | - | ENST00000301894 | ENSP00000301894 | | protein_coding | c.*593T>G | |  |  |
| 11 | 64374080 | A | C | 6 | NRXN2 | - | ENST00000301894 | ENSP00000301894 | | protein_coding | c.*588T>G | |  |  |
| 11 | 64855646 | T | G | 7 | ZFPL1 | + | ENST00000294258 | ENSP00000294258 | | protein_coding | c.*60T>G | |  |  |
| 11 | 73472123 | C | G | 6 | RAB6A | - | ENST00000336083 | ENSP00000336850 | | protein_coding | c.-443G>C | |  |  |
| 11 | 111783691 | T | C | 7 | HSPB2 | + | ENST00000304298 | ENSP00000302476 | | protein_coding | c.94+44T>C | |  |  |
| 11 | 111783691 | T | C | 7 | CRYAB | - | ENST00000526180 | ENSP00000436051 | | protein_coding | c.-290-u953A>G | | |  |
| 12 | 175968 | G | C | 7 | IQSEC3 | + | ENST00000326261 | ENSP00000315662 | | protein_coding | c.1-u81G>C | |  |  |
| 12 | 10270151 | G | A | 6 | CLEC7A | - | ENST00000396484 | ENSP00000379743 | | protein_coding | c.*390+d516C>T | | |  |
| 12 | 56388176 | A | C | 9 | RAB5B | + | ENST00000360299 | ENSP00000353444 | | protein_coding | c.*2180A>C | |  |  |
| 12 | 56551943 | A | C | 5 | MYL6 | + | ENST00000547649 | ENSP00000446714 | | protein_coding | c.-40-u203A>C | | |  |
| 12 | 56551943 | A | C | 5 | MYL6B | + | ENST00000550443 | ENSP00000446643 | | protein_coding | c.*104+d183A>C | | |  |
| 12 | 56551946 | T | C | 5 | MYL6 | + | ENST00000547649 | ENSP00000446714 | | protein_coding | c.-40-u200T>C | | |  |
| 12 | 56551946 | T | C | 5 | MYL6B | + | ENST00000550443 | ENSP00000446643 | | protein_coding | c.*104+d186T>C | | |  |
| 12 | 56632355 | A | C | 5 | ANKRD52 | - | ENST00000267116 | ENSP00000267116 | | protein_coding | c.*4571T>G | |  |  |
| 12 | 56632355 | A | C | 5 | SLC39A5 | + | ENST00000454355 | ENSP00000405360 | | protein_coding | c.*115+d725A>C | | |  |
| 12 | 117348926 | G | C | 8 | FBXW8 | + | ENST00000309909 | ENSP00000310686 | | protein_coding | c.84G>C | | cgG/cgC | p.(=) |
| 12 | 123374288 | G | A | 6 | VPS37B | - | ENST00000267202 | ENSP00000267202 | | protein_coding | c.111+6211C>T | | |  |
| 12 | 133417374 | C | G | 5 | CHFR | - | ENST00000266880 | ENSP00000266880 | | protein_coding | c.*766G>C | |  |  |
| 13 | 76111694 | G | C | 8 | COMMD6 | - | ENST00000355801 | ENSP00000348054 | | protein_coding | c.54+106C>G | | |  |
| 13 | 77455807 | G | A | 7 | KCTD12 | - | ENST00000377474 | ENSP00000366694 | | protein_coding | c.*3499C>T | |  |  |
| 14 | 20825322 | A | T | 8 | PARP2 | + | ENST00000250416 | ENSP00000250416 | | protein_coding | c.1467+14A>T | | |  |
| 14 | 22979861 | C | A | 8 | TRAJ32 | + | ENST00000390505 | ENSP00000451503 | | TR_J_gene, 5' & 3' incomplete | n.+d1471C>A | | |  |
| 14 | 24701616 | A | C | 5 | GMPR2 | + | ENST00000420554 | ENSP00000392859 | | protein_coding | c.-299-u491A>C | | |  |
| 14 | 61438247 | G | A | 9 | MNAT1 | + | ENST00000261245 | ENSP00000261245 | | protein_coding | c.*1604+d1576G>A | | |  |
| 14 | 61438247 | G | A | 9 | TRMT5 | - | ENST00000261249 | ENSP00000261249 | | protein_coding | c.*3580C>T | |  |  |
| 14 | 69726869 | A | C | 6 | GALNT16 | + | ENST00000337827 | ENSP00000336729 | | protein_coding | c.-139A>C | |  |  |
| 14 | 103399238 | C | G | 5 | AMN | + | ENST00000299155 | ENSP00000299155 | | protein_coding | c.*512+d1709C>G | | |  |
| 14 | 103399238 | C | G | 5 | CDC42BPB | - | ENST00000361246 | ENSP00000355237 | | protein_coding | c.*811G>C | |  |  |
| 14 | 105269408 | T | G | 8 | ZBTB42 | + | ENST00000555360 | ENSP00000450673 | | protein_coding | c.*20+d585T>G | | |  |
| 14 | 106354497 | T | G | 7 | IGHD2-21 | - | ENST00000390572 | ENSP00000429324 | | IG_D_gene, 5' & 3' incomplete | n.-u61A>C | |  |  |
| 14 | 106354498 | C | T | 8 | IGHD2-21 | - | ENST00000390572 | ENSP00000429324 | | IG_D_gene, 5' & 3' incomplete | n.-u62G>A | |  |  |
| 14 | 107082880 | T | G | 5 | IGHV4-59 | - | ENST00000390629 | ENSP00000375038 | | IG_V_gene, 5' & 3' incomplete | n.+d376A>C | |  |  |
| 15 | 23685448 | A | G | 5 | GOLGA6L2 | - | ENST00000312015 | ENSP00000307928 | | protein_coding | c.1633-438T>C | | |  |
| 15 | 34781861 | A | G | 7 | GOLGA8A | - | ENST00000543376 | ENSP00000438613 | | protein_coding | c.-1598+47684T>C | | |  |
| 15 | 34781867 | T | G | 5 | GOLGA8A | - | ENST00000543376 | ENSP00000438613 | | protein_coding | c.-1598+47678A>C | | |  |
| 15 | 45118056 | A | C | 5 | CTD-2008A1.2 | - | ENST00000558556 | | | transcribed_unprocessed_pseudogene | n.+d1202T>G | | |  |
| 15 | 45366326 | C | T | 5 | SORD | + | ENST00000267814 | ENSP00000267814 | | protein_coding | c.*598C>T | |  |  |
| 15 | 45366327 | A | G | 5 | SORD | + | ENST00000267814 | ENSP00000267814 | | protein_coding | c.*599A>G | |  |  |
| 15 | 45366339 | G | T | 8 | SORD | + | ENST00000267814 | ENSP00000267814 | | protein_coding | c.*611G>T | |  |  |
| 15 | 49283053 | T | G | 8 | SECISBP2L | - | ENST00000261847 | ENSP00000261847 | | protein_coding | c.*1388A>C | |  |  |
| 15 | 78288257 | G | C | 6 | TBC1D2B | - | ENST00000300584 | ENSP00000300584 | | protein_coding | c.*2245C>G | |  |  |
| 16 | 1256939 | A | C | 7 | CACNA1H | + | ENST00000348261 | ENSP00000334198 | | protein_coding | c.2790-62A>C | | |  |
| 16 | 2949264 | C | T | 5 | FLYWCH2 | + | ENST00000396958 | ENSP00000380159 | | protein_coding | c.*114C>T | |  |  |
| 16 | 7743418 | C | T | 9 | RBFOX1 | + | ENST00000355637 | ENSP00000347855 | | protein_coding | c.1111+49C>T | | |  |
| 16 | 21667506 | A | C | 7 | METTL9 | + | ENST00000358154 | ENSP00000350874 | | protein_coding | c.*753A>C | |  |  |
| 16 | 30199170 | T | G | 6 | CORO1A | + | ENST00000219150 | ENSP00000219150 | | protein_coding | c.861+20T>G | | |  |
| 16 | 31405481 | G | A | 5 | ITGAD | + | ENST00000389202 | ENSP00000373854 | | protein_coding | c.32-76G>A | |  |  |
| 16 | 32890081 | C | T | 11 | SLC6A10P | - | ENST00000330048 | | | processed_transcript | n.3472+65G>A | | |  |
| 16 | 32896688 | G | A | 10 | SLC6A10P | - | ENST00000330048 | | | processed_transcript | n.-u225C>T | |  |  |
| 16 | 33783150 | G | A | 10 | RP11-598D12.4 | + | ENST00000568600 | | | unprocessed_pseudogene | n.801+31G>A | | |  |
| 16 | 33784595 | C | G | 6 | RP11-598D12.4 | + | ENST00000568600 | | | unprocessed_pseudogene | n.1482+32C>G | | |  |
| 16 | 33784600 | T | G | 9 | RP11-598D12.4 | + | ENST00000568600 | | | unprocessed_pseudogene | n.1482+37T>G | | |  |
| 16 | 33965646 | G | A | 7 | RNA5-8SP2 | + | ENST00000363564 | | | rRNA | n.+d69G>A | |  |  |
| 16 | 33965669 | C | G | 7 | RNA5-8SP2 | + | ENST00000363564 | | | rRNA | n.+d92C>G | |  |  |
| 16 | 48387999 | T | G | 10 | LONP2 | + | ENST00000285737 | ENSP00000285737 | | protein_coding | c.*2286T>G | |  |  |
| 16 | 48388013 | T | G | 8 | LONP2 | + | ENST00000285737 | ENSP00000285737 | | protein_coding | c.*2300T>G | |  |  |
| 16 | 48388018 | C | G | 10 | LONP2 | + | ENST00000285737 | ENSP00000285737 | | protein_coding | c.*2305C>G | |  |  |
| 16 | 48388022 | T | G | 7 | LONP2 | + | ENST00000285737 | ENSP00000285737 | | protein_coding | c.*2309T>G | |  |  |
| 16 | 58700298 | G | T | 8 | SLC38A7 | - | ENST00000219320 | ENSP00000219320 | | protein_coding | c.*991C>A | |  |  |
| 16 | 70208065 | T | C | 6 | CLEC18C | + | ENST00000569347 | ENSP00000455920 | | protein_coding | c.-117T>C | |  |  |
| 17 | 7321016 | G | C | 6 | NLGN2 | + | ENST00000302926 | ENSP00000305288 | | protein_coding | c.2406G>C | | ccG/ccC | p.(=) |
| 17 | 14139075 | T | C | 5 | CDRT15 | - | ENST00000420162 | ENSP00000402355 | | protein_coding | c.*98A>G | |  |  |
| 17 | 18585042 | C | G | 5 | ZNF286B | - | ENST00000545289 | ENSP00000461413 | | protein_coding | c.-78G>C | |  |  |
| 17 | 20768761 | C | T | 7 | CCDC144NL | - | ENST00000327925 | ENSP00000328054 | | protein_coding | c.633G>A | | aaG/aaA | p.(=) |
| 17 | 36886025 | G | T | 5 | MLLT6 | + | ENST00000325718 | ENSP00000316426 | | protein_coding | c.*4174G>T | |  |  |
| 17 | 36886025 | G | T | 5 | CISD3 | + | ENST00000439660 | ENSP00000391402 | | protein_coding | c.-124-u463G>T | | |  |
| 17 | 36886025 | G | T | 5 | AC006449.1 | - | ENST00000595377 | ENSP00000470796 | | protein_coding | c.1-u1574C>A | | |  |
| 17 | 38632482 | T | G | 5 | TNS4 | - | ENST00000254051 | ENSP00000254051 | | protein_coding | c.*1358A>C | |  |  |
| 17 | 38632488 | T | G | 5 | TNS4 | - | ENST00000254051 | ENSP00000254051 | | protein_coding | c.*1352A>C | |  |  |
| 17 | 38632493 | T | G | 6 | TNS4 | - | ENST00000254051 | ENSP00000254051 | | protein_coding | c.*1347A>C | |  |  |
| 17 | 38632499 | T | G | 5 | TNS4 | - | ENST00000254051 | ENSP00000254051 | | protein_coding | c.*1341A>C | |  |  |
| 17 | 41455232 | C | T | 5 | LINC00910 | - | ENST00000341011 | | | lincRNA | n.786-51G>A | | |  |
| 17 | 46703758 | T | C | 5 | HOXB9 | - | ENST00000311177 | ENSP00000309439 | | protein_coding | c.-127A>G | |  |  |
| 17 | 47755248 | A | C | 6 | SPOP | - | ENST00000393328 | ENSP00000377001 | | protein_coding | c.-189+47T>G | | |  |
| 17 | 77111772 | T | G | 6 | RBFOX3 | - | ENST00000580155 | ENSP00000463653 | | protein_coding | c.26A>C | | cAg/cCg | p.Q9P |
| 17 | 77111776 | C | G | 8 | RBFOX3 | - | ENST00000580155 | ENSP00000463653 | | protein_coding | c.22G>C | | Gcc/Ccc | p.A8P |
| 18 | 34298333 | A | C | 7 | FHOD3 | + | ENST00000257209 | ENSP00000257209 | | protein_coding | c.2547A>C | | ccA/ccC | p.(=) |
| 18 | 57567679 | T | G | 5 | PMAIP1 | + | ENST00000316660 | ENSP00000326119 | | protein_coding | c.58+212T>G | | |  |
| 18 | 74208553 | C | G | 5 | ZNF516 | - | ENST00000443185 | ENSP00000394757 | | protein_coding | c.-318-u1407G>C | | |  |
| 19 | 1061715 | G | A | 5 | ABCA7 | + | ENST00000263094 | ENSP00000263094 | | protein_coding | c.5464-66G>A | | |  |
| 19 | 1753094 | G | C | 5 | ONECUT3 | + | ENST00000382349 | ENSP00000371786 | | protein_coding | c.-568G>C |  | |  |
| 19 | 3366562 | T | G | 6 | NFIC | + | ENST00000589123 | ENSP00000465655 | | protein_coding | c.3+6879T>G | | |  |
| 19 | 4174802 | T | G | 5 | CREB3L3 | + | ENST00000078445 | ENSP00000078445 | | protein_coding | c.*1085+d1751T>G | | |  |
| 19 | 4174802 | T | G | 5 | SIRT6 | - | ENST00000337491 | ENSP00000337332 | | protein_coding | c.880A>C | | Acc/Ccc | p.T294P |
| 19 | 4174810 | C | G | 7 | CREB3L3 | + | ENST00000078445 | ENSP00000078445 | | protein_coding | c.*1085+d1759C>G | | |  |
| 19 | 4174810 | C | G | 7 | SIRT6 | - | ENST00000337491 | ENSP00000337332 | | protein_coding | c.872G>C | | cGc/cCc | p.R291P |
| 19 | 7977460 | A | C | 5 | MAP2K7 | + | ENST00000397979 | ENSP00000381066 | | protein_coding | c.*144A>C | |  |  |
| 19 | 7977473 | G | C | 9 | MAP2K7 | + | ENST00000397979 | ENSP00000381066 | | protein_coding | c.*157G>C | |  |  |
| 19 | 17444478 | C | G | 8 | ANO8 | - | ENST00000159087 | ENSP00000159087 | | protein_coding | c.217+21G>C | | |  |
| 19 | 17444482 | C | G | 8 | ANO8 | - | ENST00000159087 | ENSP00000159087 | | protein_coding | c.217+17G>C | | |  |
| 19 | 18307715 | C | G | 5 | RAB3A | - | ENST00000222256 | ENSP00000222256 | | protein_coding | c.*565G>C | |  |  |
| 19 | 18307715 | C | G | 5 | MPV17L2 | + | ENST00000599612 | ENSP00000469836 | | protein_coding | c.*687+d163C>G | | |  |
| 19 | 35863362 | G | A | 5 | GPR42 | + | ENST00000454971 | ENSP00000410925 | | protein_coding | c.*60G>A | |  |  |
| 19 | 35863559 | T | C | 5 | GPR42 | + | ENST00000454971 | ENSP00000410925 | | protein_coding | c.*257T>C | |  |  |
| 19 | 39220197 | T | G | 5 | ACTN4 | + | ENST00000252699 | ENSP00000252699 | | protein_coding | c.*125T>G | |  |  |
| 19 | 39220197 | T | G | 5 | CAPN12 | - | ENST00000328867 | ENSP00000331636 | | protein_coding | c.*293+d1002A>C | | |  |
| 19 | 41403150 | G | A | 8 | CYP2G1P | + | ENST00000252909 | | | processed_transcript | n.1647-893G>A | | |  |
| 19 | 41403155 | G | A | 8 | CYP2G1P | + | ENST00000252909 | | | processed_transcript | n.1647-888G>A | | |  |
| 19 | 43877751 | C | T | 7 | CD177P1 | - | ENST00000378007 | | | unprocessed_pseudogene | n.+d1909G>A | | |  |
| 19 | 43877759 | C | A | 6 | CD177P1 | - | ENST00000378007 | | | unprocessed_pseudogene | n.+d1901G>T | | |  |
| 19 | 43877760 | T | C | 8 | CD177P1 | - | ENST00000378007 | | | unprocessed_pseudogene | n.+d1900A>G | | |  |
| 19 | 43877763 | A | T | 7 | CD177P1 | - | ENST00000378007 | | | unprocessed_pseudogene | n.+d1897T>A | | |  |
| 19 | 43877764 | G | T | 7 | CD177P1 | - | ENST00000378007 | | | unprocessed_pseudogene | n.+d1896C>A | | |  |
| 19 | 45572263 | G | A | 5 | CLASRP | + | ENST00000221455 | ENSP00000221455 | | protein_coding | c.1769-61G>A | | |  |
| 19 | 47777953 | A | G | 5 | PRR24 | + | ENST00000552360 | ENSP00000447679 | | protein_coding | c.-35-u189A>G | | |  |
| 19 | 50094891 | A | C | 5 | PRRG2 | + | ENST00000246794 | ENSP00000246794 | | protein_coding | c.*626+d619A>C | | |  |
| 19 | 54486426 | C | G | 8 | CACNG8 | + | ENST00000270458 | ENSP00000270458 | | protein_coding | c.*323C>G | |  |  |
| 19 | 54486431 | C | G | 7 | CACNG8 | + | ENST00000270458 | ENSP00000270458 | | protein_coding | c.*328C>G | |  |  |
| 19 | 58071007 | C | G | 6 | ZNF550 | - | ENST00000325134 | ENSP00000446224 | | protein_coding | c.27+17G>C | |  |  |
| 20 | 1553416 | G | C | 5 | SIRPB1 | - | ENST00000381605 | ENSP00000371018 | | protein_coding | c.434-733C>G | | |  |
| 20 | 5171018 | A | T | 7 | CDS2 | + | ENST00000460006 | ENSP00000419879 | | protein_coding | c.*138A>T | |  |  |
| 20 | 25667136 | T | G | 5 | ZNF337 | - | ENST00000252979 | ENSP00000252979 | | protein_coding | c.-49-34A>C | |  |  |
| 20 | 30467813 | T | G | 7 | TTLL9 | + | ENST00000375938 | ENSP00000365105 | | protein_coding | c.70-7186T>G | | |  |
| 20 | 50213003 | G | A | 6 | ATP9A | - | ENST00000338821 | ENSP00000342481 | | protein_coding | c.*4697+d50C>T | | |  |
| 20 | 58179961 | C | T | 6 | PHACTR3 | + | ENST00000359926 | ENSP00000353002 | | protein_coding | c.109+27289C>T | | |  |
| 21 | 17102405 | G | C | 6 | USP25 | + | ENST00000285679 | ENSP00000285679 | | protein_coding | c.-308G>C | |  |  |
| 21 | 42218491 | G | C | 5 | DSCAM | - | ENST00000400454 | ENSP00000383303 | | protein_coding | c.43+54C>G | |  |  |
| 21 | 45649509 | C | T | 7 | ICOSLG | - | ENST00000407780 | ENSP00000384432 | | protein_coding | c.898+428G>A | | |  |
| 21 | 46644411 | T | C | 8 | ADARB1 | + | ENST00000389863 | ENSP00000374513 | | protein_coding | c.2137-1051T>C | | |  |
| 21 | 46644496 | A | G | 9 | ADARB1 | + | ENST00000389863 | ENSP00000374513 | | protein_coding | c.2137-966A>G | | |  |
| 22 | 37764928 | A | C | 5 | ELFN2 | - | ENST00000402918 | ENSP00000385277 | | protein_coding | c.*4184T>G | |  |  |
| 22 | 37764932 | A | C | 7 | ELFN2 | - | ENST00000402918 | ENSP00000385277 | | protein_coding | c.*4180T>G | |  |  |
| 22 | 37764936 | A | C | 7 | ELFN2 | - | ENST00000402918 | ENSP00000385277 | | protein_coding | c.*4176T>G | |  |  |
| 22 | 42310611 | C | G | 5 | SHISA8 | - | ENST00000457093 | ENSP00000389964 | | protein_coding, 5' & 3' incomplete | n.-u41G>C | |  |  |
| 22 | 50927773 | T | G | 8 | MIOX | + | ENST00000216075 | ENSP00000216075 | | protein_coding | c.586+49T>G | | |  |
| X | 200424 | C | T | 6 | PLCXD1 | + | ENST00000381657 | ENSP00000371073 | | protein_coding | c.-21-410C>T | | |  |
| X | 200525 | C | T | 9 | PLCXD1 | + | ENST00000381657 | ENSP00000371073 | | protein_coding | c.-21-309C>T | | |  |
| X | 218674 | A | G | 11 | PLCXD1 | + | ENST00000381657 | ENSP00000371073 | | protein_coding | c.*2672A>G | |  |  |
| X | 219257 | G | C | 7 | PLCXD1 | + | ENST00000381657 | ENSP00000371073 | | protein_coding | c.*3255G>C | |  |  |
| X | 219261 | C | T | 8 | PLCXD1 | + | ENST00000381657 | ENSP00000371073 | | protein_coding | c.*3259C>T | |  |  |
| X | 545924 | T | G | 5 | . |  |  |  | |  |  | |  |  |
| X | 611299 | G | A | 5 | SHOX | + | ENST00000334060 | ENSP00000335505 | | protein_coding | c.634-8221G>A | | |  |
| X | 611829 | C | G | 9 | SHOX | + | ENST00000334060 | ENSP00000335505 | | protein_coding | c.634-7691C>G | | |  |
| X | 1429155 | T | C | 9 | CSF2RA | + | ENST00000355805 | ENSP00000348058 | | protein_coding | c.*559+d328T>C | | |  |
| X | 1460554 | A | G | 11 | IL3RA | + | ENST00000331035 | ENSP00000327890 | | protein_coding | c.-38-67A>G | | |  |
| X | 1511084 | T | C | 8 | SLC25A6 | - | ENST00000381401 | ENSP00000370808 | | protein_coding | c.-182A>G | |  |  |
| X | 1511113 | A | G | 9 | SLC25A6 | - | ENST00000381401 | ENSP00000370808 | | protein_coding | c.-211T>C | |  |  |
| X | 1511328 | A | G | 7 | SLC25A6 | - | ENST00000381401 | ENSP00000370808 | | protein_coding | c.-426T>C | |  |  |
| X | 1583979 | C | T | 6 | P2RY8 | - | ENST00000381297 | ENSP00000370697 | | protein_coding | c.*393G>A | |  |  |
| X | 2137767 | T | G | 12 | DHRSX | - | ENST00000334651 | ENSP00000334113 | | protein_coding | c.*1315A>C | |  |  |
| X | 2138260 | C | T | 11 | DHRSX | - | ENST00000334651 | ENSP00000334113 | | protein_coding | c.*822G>A | |  |  |
| X | 2138783 | G | A | 8 | DHRSX | - | ENST00000334651 | ENSP00000334113 | | protein_coding | c.*299C>T | |  |  |
| X | 13732283 | C | A | 6 | TRAPPC2 | - | ENST00000359680 | ENSP00000352708 | | protein_coding | c.*243G>T | |  |  |
| X | 47835102 | G | T | 9 | ZNF182 | - | ENST00000396965 | ENSP00000380165 | | protein_coding | c.*464C>A | |  |  |
| X | 48925472 | C | G | 5 | CCDC120 | + | ENST00000376396 | ENSP00000365577 | | protein_coding | c.1717C>G | | Ccc/Gcc | p.P573A |
| X | 70280626 | A | C | 10 | SNX12 | - | ENST00000374274 | ENSP00000363392 | | protein_coding | c.*240T>G | |  |  |
| X | 70586354 | T | G | 6 | TAF1 | + | ENST00000373790 | ENSP00000362895 | | protein_coding | c.180+10T>G | | |  |
| X | 70586360 | T | G | 7 | TAF1 | + | ENST00000373790 | ENSP00000362895 | | protein_coding | c.180+16T>G | | |  |
| X | 72224151 | A | C | 8 | PABPC1L2B | + | ENST00000373521 | ENSP00000362621 | | protein_coding | c.*67A>C | |  |  |
| X | 72224162 | T | C | 5 | PABPC1L2B | + | ENST00000373521 | ENSP00000362621 | | protein_coding | c.*78T>C | |  |  |
| X | 109417593 | A | C | 5 | TMEM164 | + | ENST00000372068 | ENSP00000361138 | | protein_coding | c.*914A>C | |  |  |
| X | 109417604 | T | C | 5 | TMEM164 | + | ENST00000372068 | ENSP00000361138 | | protein_coding | c.*925T>C | |  |  |
| X | 109417611 | T | C | 5 | TMEM164 | + | ENST00000372068 | ENSP00000361138 | | protein_coding | c.*932T>C | |  |  |
| X | 148632001 | C | T | 14 | CXorf40A | + | ENST00000422892 | ENSP00000422312 | | protein_coding | c.*568+d464C>T | | |  |
| X | 148853718 | T | C | 8 | TMEM185AP1 | + | ENST00000524311 | | | unprocessed_pseudogene | n.+d689T>C | |  |  |
| X | 153775100 | C | G | 5 | G6PD | - | ENST00000393564 | ENSP00000377194 | | protein_coding | c.-9+583G>C | | |  |
| X | 155009225 | C | T | 13 | SPRY3 | + | ENST00000302805 | ENSP00000302978 | | protein_coding | c.*4825C>T | |  |  |
| X | 155009829 | C | T | 12 | SPRY3 | + | ENST00000302805 | ENSP00000302978 | | protein_coding | c.*5429C>T | |  |  |
| X | 155011276 | C | T | 8 | SPRY3 | + | ENST00000302805 | ENSP00000302978 | | protein_coding | c.*6876C>T | |  |  |
| Y | 14107438 | C | T | 5 | MXRA5P1 | + | ENST00000420610 | | | unprocessed_pseudogene | n.7346C>T | |  |  |
